# The N-terminal activation function AF-1 domain of ERα interacts directly with the C-terminal AF-2-holding ligand-binding domain to recruit the coactivator proteins

**DOI:** 10.1371/journal.pone.0312276

**Published:** 2024-10-21

**Authors:** Xiaohui Liu, Yutaka Matsuyama, Makiko Sugiyama, Keitaro Suyama, Takeru Nose, Miki Shimohigashi, Yasuyuki Shimohigashi

**Affiliations:** 1 Laboratory of Structure-Function Biochemistry, Department of Chemistry, Faculty of Science, Kyushu University, Fukuoka, Japan; 2 Department of Biotechnology and Life Sciences, Faculty of Biotechnology and Life Sciences, Sojo University, Kumamoto, Japan; 3 Faculty of Arts and Science, Kyushu University, Fukuoka, Japan; 4 Division of Biology, Department of Earth System of Science, Faculty of Science, Fukuoka University, Fukuoka, Japan; 5 Risk Science Research Institute, Fukuoka, Japan; Zhejiang Normal University, CHINA

## Abstract

Cryoelectron microscopy (cryo-EM) clarified the quaternary structure of the DNA complex of coactivator-bound estrogen receptor alpha (ER*α*), revealing the adjacency of the N-terminal domain (NTD) and C-terminal ligand-binding domain (LBD). ER*α*-NTD and LBD constitute activation function 1 (AF-1) and activation function 2 (AF-2), respectively. These domains are essential for transcription activation. Their spatial proximity was judged to be essential for ER*α* to recruit the SRC coactivator proteins. In the present study, we first evaluated untethered free ER*α*-NTD(AF-1) [residues 1–180] and its-truncated desNTD(AF-1)-ER*α* [residues 181–595] in a luciferase reporter gene assay. ER*α*-NTD(AF-1) was completely inactive, whereas desNTD(AF-1)-ER*α* exhibited 66% activity of wild-type ER*α*. Surprisingly, ER*α*-NTD(AF-1) was found to inhibit desNTD(AF-1)-ER*α* markedly. Therefore, assuming that ER*α*-NTD(AF-1) must also inhibit wild-type full-length ER*α*, we co-expressed ER*α*-NTD(AF-1) and full-length ER*α*. As expected, ER*α*-NTD(AF-1) inhibited ER*α* in a dose-dependent manner, but non-competitively for 17*β*-estradiol. When their intracellular transport was examined immunocytochemically, ER*α*-NTD(AF-1) showed a distinct translocation from the cytoplasm to the nucleus, despite being expressed solely in the cytoplasm without full-length ER*α*. This nuclear translocation was attributable to a direct interaction between ER*α*-NTD(AF-1) and full-length ER*α* consisting of the nuclear localization signal. The present results demonstrated that, in full-length ER*α*, the N-terminally tethered NTD(AF-1) domain collaborates with the C-terminal LBD(AF-2) for coactivator recruitment.

## Introduction

**Fig 1:**
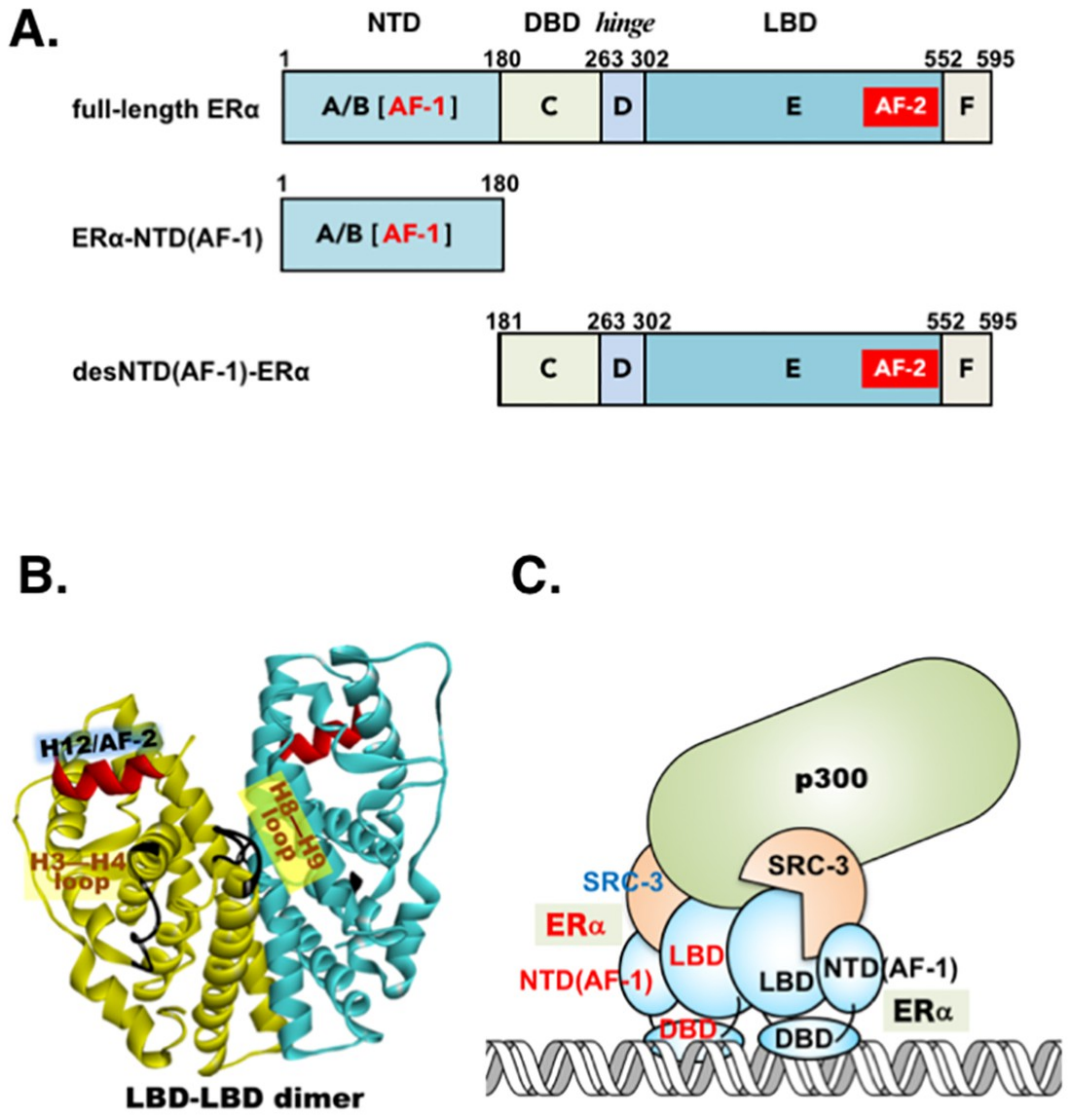
Constructive features of human estrogen receptor *α* (ER*α*). **(A)** Schematic bar chart representation of domain structures A–F of ER*α*, ER*α*-NTD(AF-1), and desNTD(AF-1)-ER*α*. Each domain plays a different structural/functional role. Alias names representing domain functions are N-terminal domain, also known as activation function 1 (AF-1) [NTD(AF-1)]; DNA-binding domain (DBD); and ligand-binding domain (LBD). The *hinge* region between DBD and LBD is part of the flexible linker. **(B)** Ordinary 3D schematic ribbon diagram representation of ER*α*-LBD in the C2 symmetric structure. *α*-Helix No. 12 (H12) (red) functions as AF-2. The original 3D structure of this dimeric representation is 1G50 (PDB identification cord), which was further analyzed using the software package DiscoveryStudio. **(C)** A symbolized cartoon illustration of the structure of the ER*α*-coactivator-DNA complex. For this complex, an annotated cryo-EM density map and its illustration were reported by Yi et al. (2015) [5]. SRC-3 is a p160 family transcriptional coactivator protein, and p300 is a secondary coactivator.

Estrogen receptor alpha (ER*α*) is a nuclear receptor (NR) that pleiotropically regulates various physiological processes, including reproduction, development, embryogenesis, and metabolism. It is activated by the sex hormone 17*β*-estradiol (E2) [1–4]. ER*α* consists of 595 amino acid residues and features a unique domain structure, similar to those of NRs, encompassing the N-terminal domain (NTD: amino acid residues 1–180), the DNA-binding domain (DBD: 181–263), and the ligand-binding domain (LBD: 303–595 including the F-domain) (Fig 1A). There is a flexible hinge linker region (*hinge*: 264–302) between DBD and LBD, and these domains are designated alphabetically A/B, C, D, E, and F arranged from the N-terminus to the C-terminus (Fig 1A). E2 binds to a specific site in the ligand-binding pocket (LBP) located within the LBD, which is in a dimeric structure (Fig 1B).

In the agonist-bound structure, *α*-helix No. 12 (H12) in LBD binds to the cavity of the pocket LBP, forming a lid over the top of the pocket. As a result of this binding, H12 creates the hydrophobic coactivator-binding site on the molecular surface. This is the reason why H12 is called activation function 2 (AF-2). The p160 family transcriptional coactivator proteins (SRC-1, SRC-2, and SRC-3, where SRC means steroid receptor coactivator) interact with H12/AF-2 via their LXXLL amino acid motifs [6–8]. This direct interaction between H12/AF-2 and the SRC coactivator protein is involved in regulating almost all aspects of gene expression [9, 10]. However, when antagonists such as 4-hydroxytamoxifen and raloxifene bind to ER*α*, their bulky side chains block H12 sterically from an agonist position [11].

In contrast to the structural or functional role of AF-2, that of activation function 1 (AF-1), which is another name for the domain NTD referred to as NTD(AF-1) hereafter (Fig 1A), has never been elucidated in detail. Using cryoelectron microscopy (cryo-EM), Yi et al. (2015) determined for the first time the quaternary structure of an active complex of DNA-bound ER*α* [5]. On a DNA molecule, the complex was found to consist of two molecules of ER*α*, two molecules of SRC-3, and one molecule of the secondary coactivator p300 (Fig 1C). The dimeric structure of ER*α*-LBD confirms the composition of this complex (Fig 1B). Through density map analysis using an ER*α*-NTD(AF-1)-specific monoclonal antibody, they concluded that the N-terminal ER*α*-NTD(AF-1) domain is adjacent to ER*α*-LBD holding AF-2, called LBD(AF-2) hereafter (Fig 1C). The close proximity of NTD(AF-1) and LBD(AF-2) was judged to be a structure essential for recruiting the SRC-3 coactivator protein [5].

In the primary structure of full-length ER*α*, N-terminal NTD(AF-1) and C-terminal LBD(AF-2) are apart from each other (Fig 1A). The tethering structure that NTD(AF-1) is linked to the central DBD domain must be crucial for the formation of a binding site for SRC-3. In the present study, to evaluate the intrinsic significance of positioning AF-1 at the ER*α* N-terminus, we tested a synthetic untethered free ER*α*-NTD(AF-1) for its-truncated desNTD(AF-1)-ER*α* and full-length ER*α*. Here we discuss the molecular mechanisms underlying the function of ER*α*-NTD(AF-1) as a transcriptional activation factor.

## Materials and methods

### Construction of ER*α*, ER*α*-NTD(AF-1), and desNTD(AF-1)-ER*α* plasmids

The expression plasmid full-length ER*α*/pcDNA3.1 of wild-type full-length ER*α* was generated by PCR using an ER*α* cDNA clone (OriGene Technologies, Rockville, MD, USA) followed by cloning into the vector pcDNA3.1(+) (Invitrogen, Carlsbad, CA, USA), as reported [12, 13]. Using full-length ER*α* as a template, expression plasmids ER*α*-NTD(AF-1)/pcDNA3.1 and desNTD(AF-1)-ER*α*/pcDNA3.1 were generated by PCR followed by cloning into pcDNA3.1(+).

For the immunocytochemical study, the NTD(AF-1) domain was modified at the N-terminus to be tagged with DYKDDDDK, a FLAG-epitope peptide [14], yielding the plasmid FLAG-ER*α*-NTD(AF-1)/pcDNA3.1. The expression plasmid full-length ER*β*/pcDNA3.1 of wild-type ER*β* was generated by PCR using an ER*β* cDNA clone. Using this as a template, FLAG-ER*β*-NTD(AF-1)/pcDNA3.1 was prepared similarly. All plasmid constructs were confirmed by DNA sequencing.

### Cell culture and luciferase reporter gene assay

HeLa cells (RCB0007; RIKEN BRC, Tsukuba, Japan) were seeded at 5 × 10^5^ cells/well of a 6-well microplate for 24 h at 37°C in a 5% CO_2_ atmosphere, as reported [15–18], using modified Eagle’s Minimum Essential Medium (Nissui, Tokyo, Japan) in the presence of 10% (v/v) charcoal-treated fetal bovine serum. The following three plasmids were co-transfected in the total 2.0 mL medium: (i) firefly luciferase reporter plasmid pGL3 (Promega, Madison, WI, USA) containing a triple-repeat estrogen response element (3 × ERE/pGL3; ERE = AGGTCAnnnTGACCT), (ii) expression plasmid full-length ER*α*/pcDNA3.1, and (iii) pGL4.74 (Promega) internal control plasmid containing the *Renilla* luciferase cDNA [19]. The transfection was performed with Lipofectamine^*TM*^ 3000 transfection reagent (Thermo Fisher Scientific, Waltham, MA, USA) according to the manufacturer’s protocol. The transfected cells were cultured for approximately 24 h at 37°C in a 5% CO_2_ atmosphere.

For the luciferase reporter gene assay, the cells were harvested using 0.25% trypsin/0.02% EDTA solution and then plated onto 96-well microplates at 5 × 10^4^ cells/well. After the agonist E2 was administered at different concentrations, the cells were incubated for another 24 h. The firefly/*Renilla* luciferase activities were measured and normalized using a dual-luciferase reporter assay system (Promega). Light emission was measured on a Fluoroskan FL microplate reader (Thermo Fisher Scientific). Each assay was performed in duplicate at least three times.

To evaluate the transcription activity of desNTD(AF-1)-ER*α*, expression plasmid desNTD(AF-1)-ER*α*/pcDNA3.1 was used instead of full-length ER*α*/pcDNA3.1. To assess the effect of ER*α*-NTD(AF-1) on desNTD(AF-1)-ER*α*, the expression plasmids ER*α*-NTD(AF-1)/pcDNA3.1 and desNTD(AF-1)-ER*α*/pcDNA3.1 were co-transfected into the cells simultaneously at the ratio of X: 1, where X [ER*α*-NTD(AF-1)] = 1, 2, 3, 4, 5, and 6, with the fixed net amount of desNTD(AF-1)-ER*α* = 250 ng/well. The luciferase reporter gene assays were carried out exactly as described above.

### Co-expression of ER*α*-NTD(AF-1) and full-length ER*α* or ER*β*

#### Gene transfection

An untethered free ER*α*-NTD(AF-1) fragment (1–180) and ER*α*-NTD(AF-1) or epitope-modified FLAG-ER*α*-NTD(AF-1) were tested for their ability to affect the transcription activity of E2 for full-length ER*α* (1–595). Test solutions were prepared using 6-well microplates, delivering HeLa cells (5 × 10^5^ cells) into each well. Expression plasmid ER*α*-NTD(AF-1)/pcDNA3.1 or FLAG-ER*α*-NTD(AF-1)/pcDNA3.1 was first transfected, administered in a dose-dependent manner (0.01–2.00 *μ*g/well) (Table 1). The total amount of the expression vector and plasmids was maintained at 2.50 *μ*g/well to utilize the Lipofectamine^*TM*^ 3000 transfection reagent (Thermo Fisher Scientific), and this was adjusted to 0.25–2.25 *μ*g/well of the pcDNA3.1 empty vector for each transfection (Table 1). Other expression plasmids were used in fixed amounts: full-length ER*α*/pcDNA3.1 (0.05 *μ*g/well), 3 × ERE/pGL3 (0.15 *μ*g/well), and pGL4.74 (0.05 *μ*g/well) (Table 1).

**Table 1:** Preparation of test solutions for ER*α*-NTD(AF-1) inhibition assay: Amount of introduced plasmid genes for a single well of the 6-well cell culture microplate.

	Amount of each gene introduced (µg/well^1)^)															
Plasmids	I	II	III	IV	V	VI	VII	VIII	IX	X	XI	XII	XIII	XIV	XV^2)^	XVI^3)^
ER*α*-full^4)^/pcDNA3.1	0.05	0.05	0.05	0.05	0.05	0.05	0.05	0.05	0.05	0.05	0.05	0.05	0.05	0.05	0.00	0.00
3 × ERE/pGL3	0.15	0.15	0.15	0.15	0.15	0.15	0.15	0.15	0.15	0.15	0.15	0.15	0.15	0.15	0.15	0.15
ER*α*-NTD(AF-1)/pcDNA3.1	0.00	0.01	0.02	0.05	0.10	0.20	0.40	0.80	1.00	1.20	1.40	1.60	1.80	2.00	0.10	0.00
pcDNA3.1^5)^	2.25	2.24	2.23	2.20	2.15	2.05	1.85	1.45	1.25	1.05	0.85	0.65	0.45	0.25	2.20	2.30
pGL4.74^6)^	0.05	0.05	0.05	0.05	0.05	0.05	0.05	0.05	0.05	0.05	0.05	0.05	0.05	0.05	0.05	0.05
**Total DNA** ^7)^	**2.50**	**2.50**	**2.50**	**2.50**	**2.50**	**2.50**	**2.50**	**2.50**	**2.50**	**2.50**	**2.50**	**2.50**	**2.50**	**2.50**	**2.50**	**2.50**

^1)^ For the gene transfection, a 6-well microplate was used for cell culture. 5 × 10^5^ HeLa cells were delivered into each well (final volume: 2 mL).

^2)^ Under the condition XV and XVI with no full-length ER*α*, it is necessary to assure that the expression plasmids of ER*α*-NTD(AF-1)/pcDNA3.1 and pcDNA3.1 do not bind to the estrogen response element (ERE).

^3)^ Under the condition with no full-length ER*α*, it is necessary to assess the basal activity derived from the endogenous ER*α*.

^4)^ The abbreviation of ‘full-length ER*α*’.

^5)^ Empty plasmid vector which does not contain DNA of either full-length ER*α* or ER*α*-NTD(AF-1).

^6)^ The vector encoding the luciferase reporter gene *hRluc* (*Renilla reniformis*).

^7)^ The limitation of total DNA amount, depending on the consumption of Lipofectamine^*TM*^ 3000 transfection reagent.

#### Luciferase reporter gene assay

Approximately 24 h after the gene transfection, cells were harvested and plated onto 96-well microplates at 5 × 10^4^ cells/well. The transcription activity at each dose was assessed for three E2 concentrations (0.1, 1, and 50 nM).

#### Assay of ER*β*-NTD(AF-1) for ER*α*

To evaluate the specificity of ER*α*-NTD(AF-1) for full-length ER*α*, co-expression tests were carried out using the plasmids containing full-length ER*α*/pcDNA3.1 and FLAG-ER*β*-NTD(AF-1)/pcDNA3.1. The assay was performed in duplicate at least three times under the conditions described above.

### Detailed inhibition tests of ER*α*-NTD(AF-1)

Gene transfections followed by luciferase reporter gene assays were carried out basically as described above. Test solutions were prepared using 6-well microplates with HeLa cells (5 × 10^5^ cells/well). First, for a detailed qualitative analysis of the inhibitory activity of non-tagged ER*α*-NTD(AF-1), an examination was carried out first by cellular transfection with serial amounts (0.01–2.00 *μ*g/well) of the plasmid ER*α*-NTD(AF-1)/pcDNA3.1 as reported previously [20]. Further transfections were carried out using the following plasmids: full-length ER*α*/pcDNA3.1 (0.10 *μ*g/well), 3 × ERE/pGL3 (0.30 *μ*g/well), pcDNA3.1 (0.00–1.99 *μ*g/well), and pGL4.74 (0.10 *μ*g/well). After cell preparation, a luciferase reporter gene assay for 10 nM E2 was conducted on a 96-well microplate using the gene-transfected cells (5 × 10^4^ cells/well). The assessed activity of E2 was plotted for the original transfection amount (g) of ER*α*-NTD(AF-1) plasmid.

Another detailed quantitative analysis of the inhibitory action of ER*α*-NTD(AF-1) was performed, again following the previously reported method [20]. Using a 6-well microplate with HeLa cells (5 × 10^5^ cells/well), the expression plasmid of ER*α*-NTD(AF-1)/pcDNA3.1 was transfected at nine concentrations: 10, 31.6, 100, 316, 500, 1000, 1200, 1600, and 2000 ng/well, together with other expression plasmids of full-length ER*α*/pcDNA3.1 (0.05 *μ*g/well), 3 × ERE/pGL3 (0.15 *μ*g/well), pcDNA3.1 (0.25–2.25 *μ*g/well), and pGL4.74 (0.05 *μ*g/well). The luciferase reporter gene assay was conducted using a 96-well microplate containing gene-transfected cells (5 × 10^4^ cells/well), and the activity of E2 (1.0×10^−13^– 1.0 × 10^−6^ M) was examined for full-length ER*α*.

From the dose‒response curves of E2, different maximal activity levels were attained, depending on the transfection amounts of ER*α*-NTD(AF-1). Thus, each distinct maximal activity was evaluated precisely as a percentage. To further analyze the inhibitory activity, the maximal activities (%) were assessed based on the original transfection amount (g) of the ER*α*-NTD(AF-1) plasmid.

### Statistical analysis

All data are shown as means ± SD for the indicated number of separate experiments. The statistical significance of differences was determined by a two-sided Student’s *t*-test. P-values less than 0.001 were considered significant.

### Immunocytochemical confocal microscopy analysis

To observe the locations of FLAG-tagged ER*α*-NTD(AF-1) and/or wild-type full-length ER*α* in the HeLa cells, they were expressed simultaneously or independently. FLAG-ER*α*-NTD(AF-1) was monitored with anti-FLAG antibody, while full-length intact ER*α* was monitored directly with ER*α*-specific antibody.

Cells were cultured in an Imaging Chamber CG 8 Well (Zell-Kontakt GmbH; Nörten-Hardenberg, Germany) at 37°C under 5% CO_2_ for 48 h after transient transfection of the full-length ER*α*/pcDNA3.1 plasmid with or without the expression plasmid of FLAG-ER*α*-NTD(AF-1)/pcDNA3.1. For immunocytochemical labeling at 25°C, cells were first washed with phosphate-buffered saline (PBS) twice, then fixed with 4% paraformaldehyde and permeabilized using 0.2% Triton X-100 in PBS for 20 min. Cells were then washed in PBS three times for 10 min each time.

After blocking with 2% BSA and 0.1% Tween 20 in PBS overnight at 4°C, the cells were incubated with anti-FLAG antibody produced in a rabbit (Sigma-Aldrich, St. Louis, MO, USA) at a 1:800 dilution and anti-ER*α* (C-311) mouse monoclonal antibody IgG_2a_ (Santa Cruz Biotechnology, Dallas, TX, USA) at a 1:1000 dilution in PBS-containing 2% BSA and 0.1% Tween 20 for 2 h. After washing with PBS three times for 10 min each time, the cells were further incubated for 60 min with Alexa Fluor 488-conjugated goat anti-rabbit IgG(H+L) secondary antibody (Thermo Fisher Scientific) (1:1000) for FLAG-ER*α*-NTD(AF-1), or Cy3-conjugated goat anti-mouse IgG(H+L) secondary antibody (Thermo Fisher Scientific) (1:1000) for full-length ER*α*. Finally, the cells were observed with an ECLIPSE TE2000-U confocal microscope (Nikon, Tokyo, Japan). A series of cellular treatments was conducted in an Imaging Chamber CG 8 Well to prepare cells for the cell culture, immunofluorescence staining, and imaging with a removable 8-well chamber.

## Results

### Activities of truncated ER*α* derivatives in the luciferase reporter gene assay

**Fig 2:**
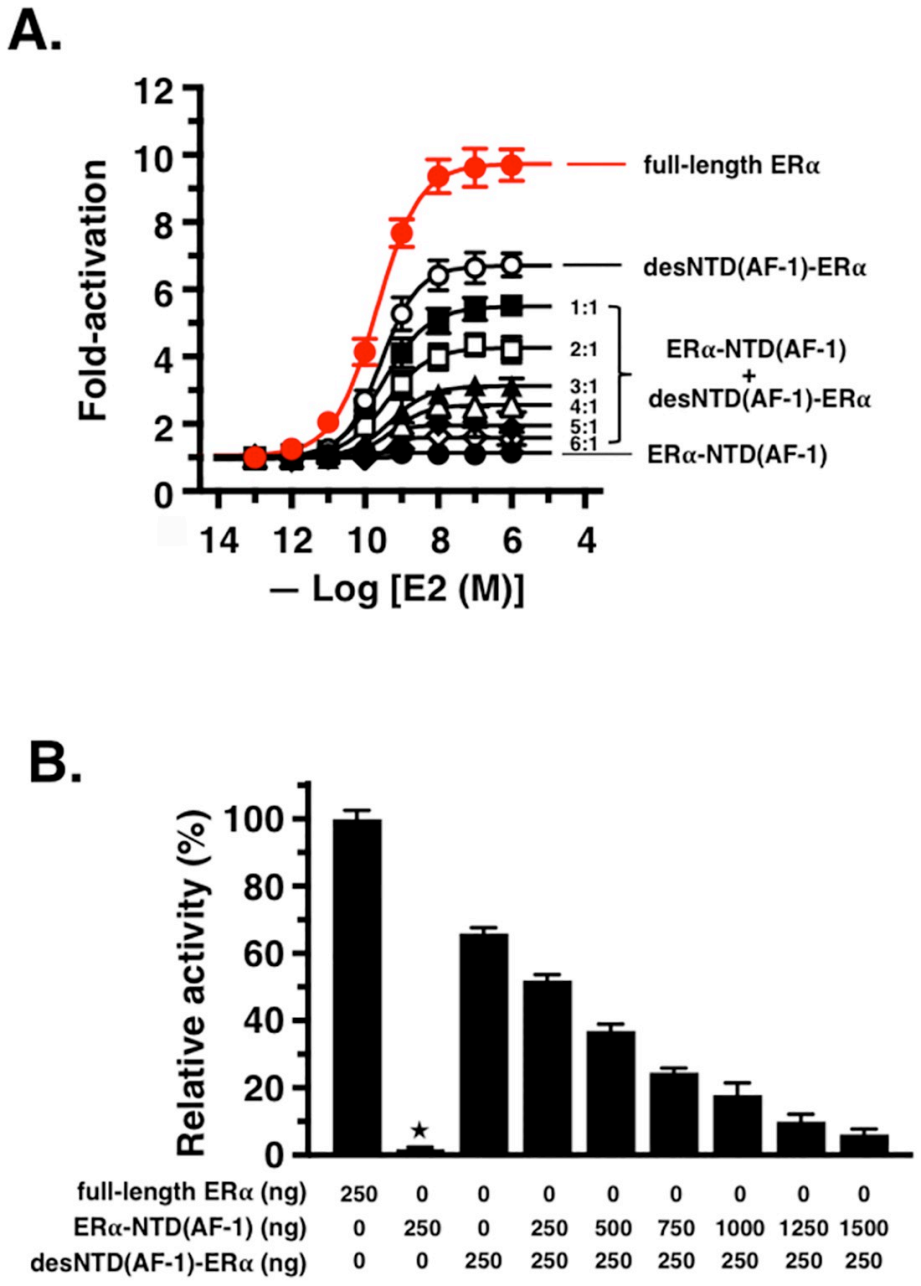
Effects of co-expression of ER*α*-NTD(AF-1) and desNTD(AF-1)-ER*α* on E2 activity in the luciferase reporter gene assay. **(A)** Dose–response curves for the activity of E2 (1.0 × 10^−13^– 1.0 × 10^−6^ M) in HeLa cells transfected with expression plasmids of full-length ER*α* (•), desNTD(AF-1)-ER*α* (◯), and ER*α*-NTD(AF-1) (•). desNTD(AF-1)-ER*α* activity was further evaluated in the presence of ER*α*-NTD(AF-1) under six different transfection conditions: with expression plasmid ratios of ER*α*-NTD(AF-1): desNTD(AF-1)-ER*α* = 1:1 (■), 2:1 (▫), 3:1 (▲), 4:1 (△), 5:1 (♦), and 6:1 (◇). **(B)** Varied maximal activity (%) of E2 for desNTD(AF-1)-ER*α* shown by the bar graphs. Relative activities of full-length ER*α* and ER*α*-NTD(AF-1) were 100% and 0% (⋆), respectively, with a transfected plasmid amount of 250 ng/well in the 6-well microplate used for transfection.

Dividing ER*α* into two parts—the N-terminal ER*α*-NTD(AF-1) and the C-terminal desNTD(AF-1)-ER*α* (Fig 1A)—we tested both in the luciferase reporter gene assay. Luciferase activity in the HeLa cells was measured approximately 48 h after their transfection. ER*α*-NTD(AF-1) was found to be completely devoid of transcription activation and exhibited absolutely no response to the ER*α* agonist 17*β*-estradiol (E2) (Fig 2). On the other hand, desNTD(AF-1)-ER*α* exhibited a definite transcription activation, although it was distinctly lower (approximately 34% lower) than the activity of full-length ER*α* (Fig 2B). The half-maximal effective concentration (EC_50_) of E2 was very low, approximately 0.21 nM, showing a high activity for full-length ER*α* (Fig 2A). For desNTD(AF-1)-ER*α*, E2 also exhibited very high activity (EC_50_ = 0.29 nM).

The 1:1 co-expression of N-terminal ER*α*-NTD(AF-1) and C-terminal desNTD(AF-1)-ER*α* resulted in an approximate 20% reduction in E2-induced transactivation compared to that mediated by desNTD(AF-1)-ER*α* alone (Fig 2B). This activity reduction was further enhanced by up to 44%, 62%, 73%, 85%, and 91% with the co-expression ratios of ER*α*-NTD(AF-1): desNTD(AF-1)-ER*α* = 2:1, 3:1, 4:1, 5:1, and 6:1, respectively. The result of dose-dependent inhibition clearly demonstrated that ER*α*-NTD(AF-1) acts as an efficient inhibitor of desNTD(AF-1)-ER*α*. The apparent IC_50_ value was estimated to be 863 ng/well, where “well” indicates a well of the 6-well microplate originally used for the transfection of ER*α*-NTD(AF-1).

It is evident that the coexistence of ER*α*-NTD(AF-1) and desNTD(AF-1)-ER*α* does not lead to the amino acid sequential additivity necessary to reproduce ER*α*’s full activity. On the contrary, the co-expression of ER*α*-NTD(AF-1) seemed to interfere with desNTD(AF-1)-ER*α* in some manner within the transcription mechanism. It should be noted that this activity interference or inhibition depends on the concentration of ER*α*-NTD(AF-1), and the target of this inhibition is desNTD(AF-1)-ER*α*, which consists of the ligand-binding domain LBD having AF-2.

### Effects of ER*α*-NTD(AF-1) on ER*α* transcription activity

The fact that ER*α*-NTD(AF-1) inhibited desNTD(AF-1)-ER*α* strongly suggested that untethered free ER*α*-NTD(AF-1) interacts with LBD(AF-2) but does not facilitate the construction of the binding site(s) for SRC coactivator(s). Since this led us to assume that ER*α*-NTD(AF-1) must inhibit also the full-length ER*α*, we decided to carry out an inhibition experiment to confirm this hypothesis. Inhibitory effectiveness was assessed by administering a wide range of concentrations (0.01–2.00 *μ*g/well) of expression plasmid ER*α*-NTD(AF-1)/pcDNA3.1 or FLAG-ER*α*-NTD(AF-1)/pcDNA3.1 for full-length ER*α*/pcDNA3.1 (0.05 *μ*g/well) and E2 (0.1, 1, or 50 nM) (Table 1).

**Fig 3:**
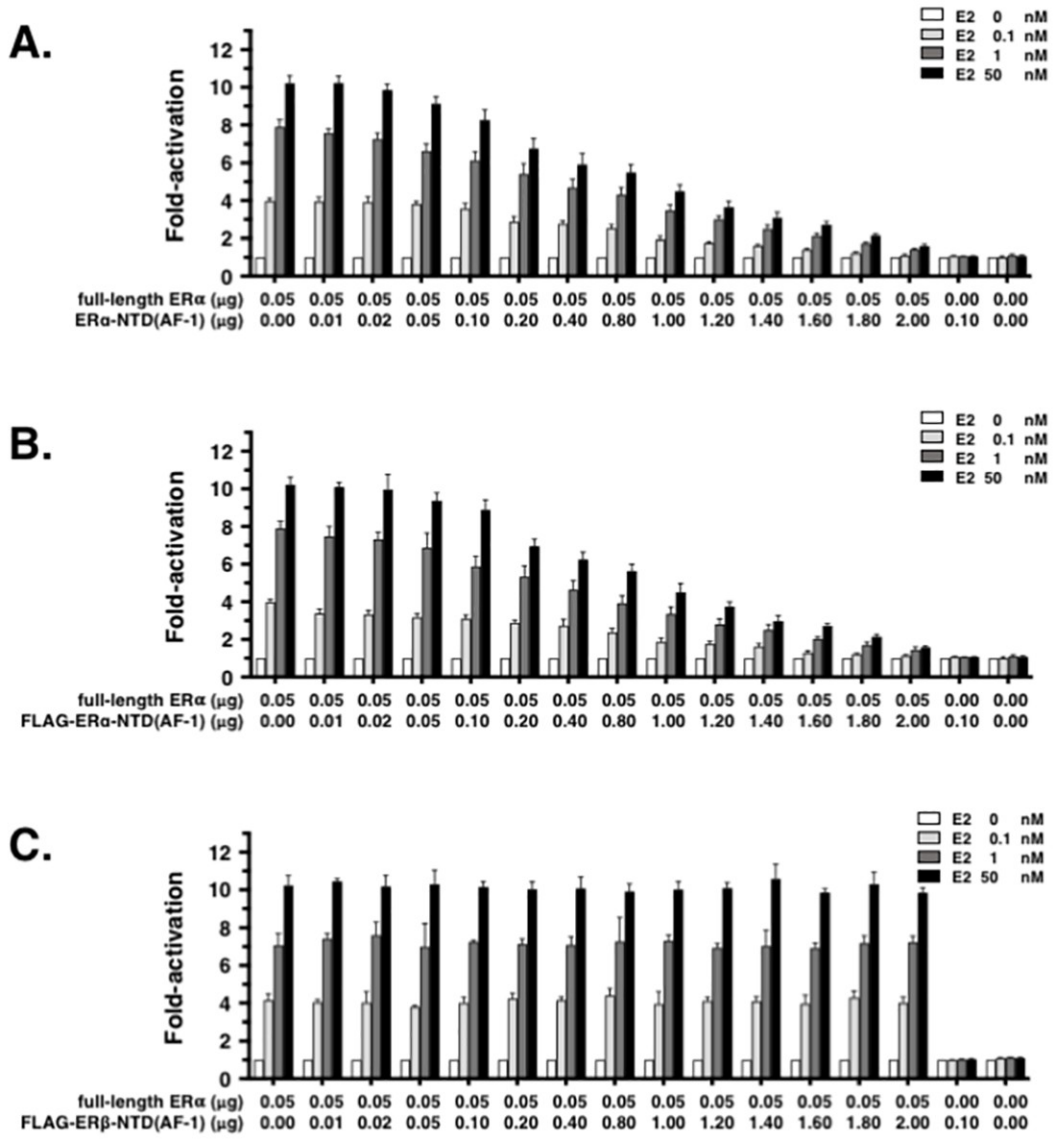
Effects of untethered free ER*α*-NTD(AF-1) on the transcription activation activity of agonist E2 and receptor full-length ER*α* or ER*β*. Luciferase reporter assays carried out for ER*α* in HeLa cells using a constant concentration of E2 (0.1, 1, or 50 nM). The effects examined were ER*α*-NTD(AF-1) **(A)** and FLAG-ER*α*-NTD(AF-1) **(B)**. Plasmid amounts used for gene transfection (ng/well of a 6-well microplate; see Table 1) are noted under the bar graphs. **(C)** Luciferase reporter assays to validate the receptor specificity of ER*α*-NTD(AF-1) for ER*α*. The NTD(AF-1) domain of another estrogen receptor, ER*β*, was tested for ER*α* in this series of assays, in which FLAG-ER*β*-NTD(AF-1) was used instead of FLAG-ER*α*-NTD(AF-1). All other assay conditions were exactly the same as in **(B)**.

This assessment revealed that the transcription activity of full-length ER*α* was reduced in a dose-dependent manner according to the transfection amounts of the ER*α*-NTD(AF-1) plasmid (Fig 3A). At the constant concentration of E2, increases in ER*α*-NTD(AF-1) gradually diminished the transcription activity of full-length ER*α*. Obviously, ER*α*-NTD(AF-1) worked to inhibit full-length ER*α*. This inhibitory activity of ER*α*-NTD(AF-1) was reproduced at all three E2 test concentrations: 0.1, 1, and 50 nM (Fig 3A). These results clearly revealed that the co-expressed synthetic ER*α*-NTD(AF-1) fragment competes with intact ER*α*-tethering NTD(AF-1), probably for the same site in ER*α*-LBD(AF-2). This competition for interaction appears to reduce the luciferase transcription activity of full-length ER*α* in a manner dependent on the amount of transfected ER*α*-NTD(AF-1).

For an immunocytochemical study to confirm the interaction between ER*α*-NTD(AF-1) and full-length ER*α*, ER*α*-NTD(AF-1) was tagged with octapeptide DYKDDDDK, named FLAG-epitope peptide, at the N-terminus [14]. To evaluate the effect of this FLAG tag peptide on inhibitory activity, FLAG-ER*α*-NTD(AF-1) was also tested in the luciferase reporter assay. It immediately became clear that FLAG-ER*α*-NTD(AF-1) inhibits full-length ER*α* and E2 as well as non-tagged free ER*α*-NTD(AF-1) (Fig 3B). The presence or absence of the FLAG-tag was found to have no impact on the inhibitory activity of ER*α*-NTD(AF-1). It should be noted that untethered free ER*α*-NTD(AF-1) possesses an intrinsic ability to inhibit full-length ER*α*.

To evaluate the specificity of ER*α*-NTD(AF-1) in receptor inhibition, the NTD(AF-1) domain of another estrogen receptor, ER*β*, was tested for full-length ER*α*. As shown in Fig 3C, FLAG-ER*β*-NTD(AF-1) had no effect on the transcription activity of ER*α*. Thus, the inhibition of full-length ER*α* is particular and unique to ER*α*-NTD(AF-1) but not to ER*β*-NTD(AF-1). The interaction between NTD(AF-1) and LBD(AF-2) in ER*α* appears to be highly specific.

### Detailed analysis of ER*α* inhibition by ER*α*-NTD(AF-1)

**Fig 4:**
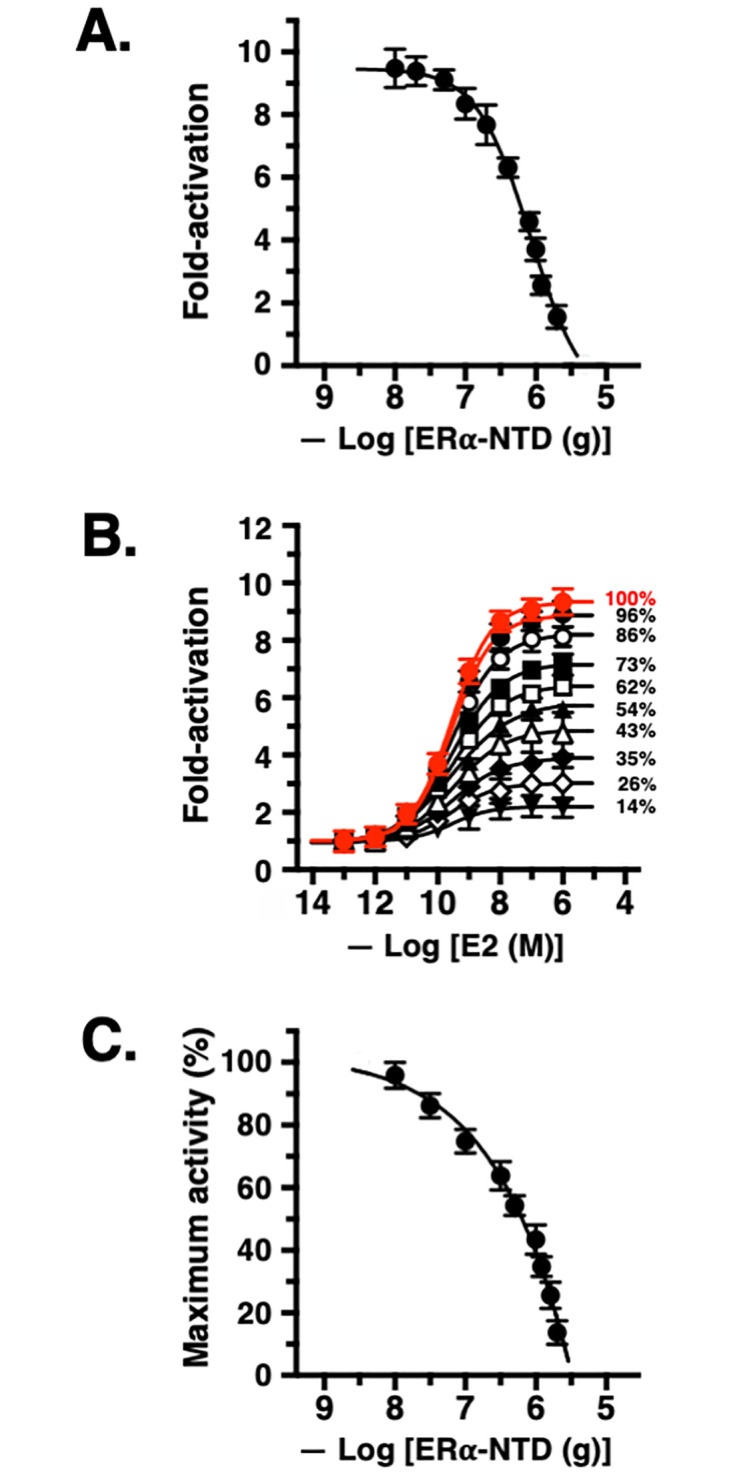
Inhibition tests in the luciferase reporter gene assay for E2 and ER*α* in the presence of ER*α*-NTD(AF-1) fragment peptide (1–180). **(A)** Dose–response curves illustrating the residual activity of 10 nM E2 in ER*α* in the presence of HeLa cells transfected with serial amounts of the expression plasmid encoding ER*α*-NTD(AF-1). **(B)** Varied maximal activity of E2 (1.0 × 10^−13^–1.0 × 10^−6^ M) in the absence (•) and presence of nine different transfection amounts of the ER*α*-NTD(AF-1) plasmid [10 (•)), 31.6 (◯), 100 (■), 316 (▫), 500 (▲), 1000 (△), 1200 (♦), 1600 (◇), and 2000 (▼) ng/well of the 6-well microplate used for transfection], and **(C)** dose–response curve depicting the relationship between the amount of the ER*α*-NTD(AF-1) plasmid used for cellular transfection and the maximal activity of E2 in the luciferase reporter gene assay.

The inhibitory activity of ER*α*-NTD(AF-1) was examined using both a qualitative analytical method and a quantitative analytical method. First, the inhibitory activity of ER*α*-NTD(AF-1) was verified qualitatively in a luciferase reporter gene assay. Prior to the assay, HeLa cells were transfected with serial amounts of the plasmid of non-tagged free ER*α*-NTD(AF-1) (1.0 × 10^−8^ to 2.0 × 10^−6^ g/well of a 6-well microplate). The activity of 10 nM E2 was evaluated for full-length ER*α* using cells treated with these various amounts of ER*α*-NTD(AF-1). At 10 nM, E2 exhibited 90–95% transcriptional activity for ER*α*. When this amount of E2 was tested in the cells treated with ER*α*-NTD(AF-1), the activity decreased according to the amount of ER*α*-NTD(AF-1) (Fig 4A). It is evident that untethered free ER*α*-NTD(AF-1) functions to inhibit full-length ER*α*. From the dose–response curve, the apparent IC_50_ value was estimated to be 836 ng/well, equivalent to the activity obtained for ER*α*-NTD(AF-1) in the inhibition of desNTD(AF-1)-ER*α* (see above).

We next evaluated the transcriptional activity of E2 quantitatively in the presence of non-tagged free ER*α*-NTD(AF-1) across nine transfection amounts: 10, 31.6, 100, 316, 500, 1000, 1200, 1600, and 2000 ng/well (where “well” indicates a well of the 6-well microplate used for transfection). As shown in Fig 4B, each dose–response curve of E2 (1.0 × 10^−13^– 1.0 × 10^−6^ M) reached a different level of maximal activation activity. The activity levels varied depending on the amounts of ER*α*-NTD(AF-1) plasmid used for transfection, as observed for the inhibition of desNTD(AF-1)-ER*α*. When we show the activity as a percentage of the full activity (100%) of neat full-length ER*α*, the maximal activation activity level attenuated gradually from 96% to 14% (Fig 4B, Table 2). In other words, when the transfection amount increased, ER*α*-NTD(AF-1) sharply reduced the maximal activity of E2 (Fig 4B).

**Table 2:** Analyses of inhibitory activity of ER*α*-NTD(AF-1) for ER*α*/17*β*-estradiol (E2) in the luciferase reporter gene assay.

Inhibitor concentration [ER*α*-NTD(AF-1)] (ng/well)^1)^	Agonist 17*β*-estradiol (E2)	
	Maximal activity (%)	EC_50_ (nM)
0	100	0.277
10	96	0.290
31.6	86	0.289
100	76	0.297
316	62	0.309
500	54	0.298
1,000	43	0.318
1,200	35	0.308
1,600	26	0.280
2,000	14	0.273
**Average EC_50_** ^2)^	0.296	

^1)^ The reaction solutions were prepared in the well of a 6-well microplate.

^2)^ The EC_50_ values of E2, which were estimated in the presence of inhibitor ER*α*-NTD(AF-1), were averaged.

The reduction of maximal activation activity signifies a decrease in the active receptor population. In the luciferase reporter gene assays carried out for this test, E2 showed almost the same EC_50_ values (Fig 4B, Table 2). The average EC_50_ value was 0.296 nM, and the intrinsic EC_50_ value of E2 was 0.277 nM with no ER*α*-NTD(AF-1) transfection (Table 2). Together, these results implied that ER*α*-NTD(AF-1) reduces the activity of full-length ER*α* without competing with E2 itself at the binding site. Apparently, ER*α*-NTD(AF-1) diminished the proper functioning of ER*α* receptor molecules. It is evident that ER*α*-NTD(AF-1) is a kind of non-competitive inhibitor of full-length ER*α*. As the transfection amount of ER*α*-NTD(AF-1) increased, the maximal activity of ER*α*/E2 decreased.

These results made it impossible to conduct a Schild plot analysis, which is applicable only for competitive inhibitors [21, 22]. Since it was obvious that there is a dose–response relationship between the transfection amount of inhibitor ER*α*-NTD(AF-1) and the maximal activity of ER*α*/E2 (Table 2), we generated a dose–response curve to assess the IC_50_ of ER*α*-NTD(AF-1) (Fig 4C). This value was calculated to be 631 ng/well, where, as noted above, “well” refers to a well of the 6-well microplate originally used for the transfection of the ER*α*-NTD(AF-1) plasmid. This IC_50_ value was roughly equivalent to that obtained from the qualitative luciferase reporter gene assay (Fig 4A). These results demonstrated that separately synthesized ER*α*-NTD(AF-1) effectively inhibited the transcription activation activity of E2 for full-length ER*α*, but in a non-competitive manner. In conclusion, untethered free ER*α*-NTD(AF-1) is a strong inhibitor of full-length ER*α*.

### Nuclear translocation of ER*α*-NTD(AF-1) by binding to ER*α*

Separately expressed ER*α*-NTD(AF-1) exhibited strong inhibitory activity in the transcription activation mechanism of full-length ER*α*. This outcome implied that untethered free ER*α*-NTD(AF-1) binds to full-length ER*α*, probably to its C-terminal LBD. The resulting complex between the ER*α*-NTD(AF-1) fragment and full-length ER*α* abrogates ER*α* transcription, potentially halting the activity at a crucial early stage of gene expression. Subsequently, we intended to demonstrate these processes immunocytochemically by staining the ER*α*-NTD(AF-1) fragment and full-length ER*α* separately. ER*α*-NTD(AF-1) was thus tagged with FLAG-epitope peptide at the N-terminus for detection with the polyclonal anti-FLAG antibody. As demonstrated above, the presence of the FLAG-tag had no influence on the inhibitory activity of ER*α*-NTD(AF-1).

ER*α* was stained by a mouse monoclonal antibody (IgG_2*a*_) raised against ER*α*. This anti-ER*α* monoclonal antibody specifically recognizes the C-terminal region (495–595) of ER*α*. Therefore, the separately synthesized N-terminal fragment ER*α*-NTD(AF-1) does not respond to this antibody. In fact, it was confirmed in advance that the anti-FLAG antibody and anti-human ER*α* antibody do not cross-react at all between ER*α*-NTD(AF-1) and full-length ER*α*.

**Fig 5:**
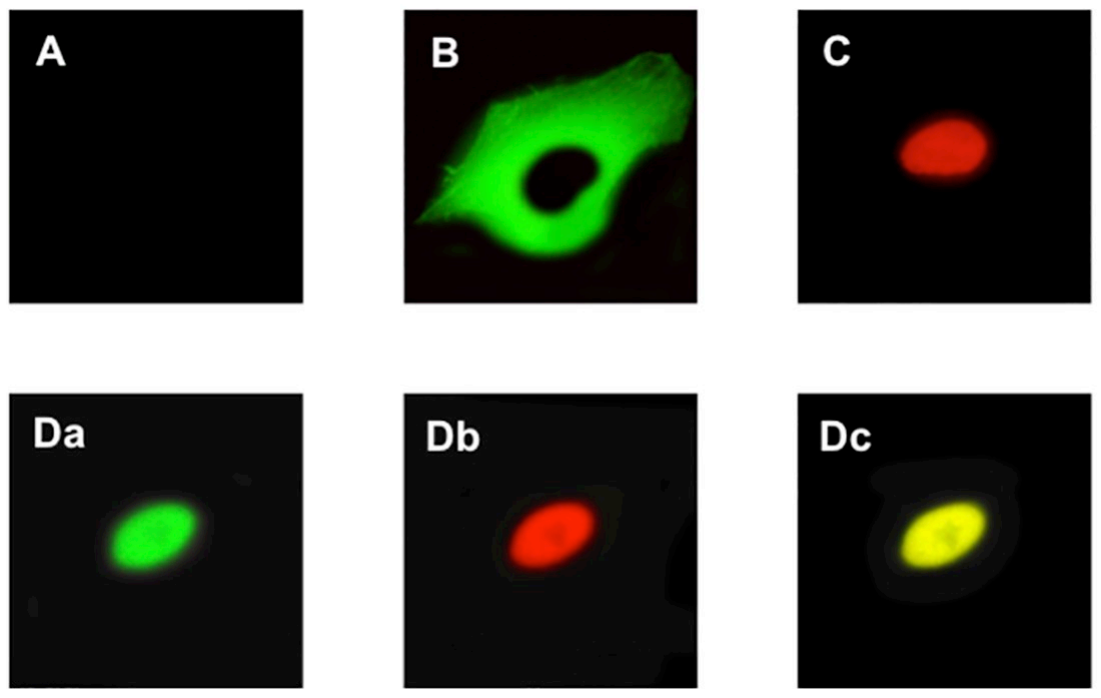
Immunocytochemical localization of FLAG-ER*α*-NTD(AF-1) and full-length ER*α* in HeLa cells. **(A)** Control staining with rabbit polyclonal anti-FLAG primary antibody, followed by Alexa Fluor 488-conjugated goat anti-rabbit IgG(H+L) secondary antibody for FLAG-ER*α*-NTD(AF-1). Similar control staining was performed using anti-ER*α* mouse monoclonal primary antibody and Cy3-conjugated goat anti-mouse IgG(H+L) secondary antibody, which were used for full-length ER*α* (data not shown). **(B)** Only FLAG-ER*α*-NTD(AF-1) was expressed in the cells. The peptide was detected as a green color. **(C)** Only full-length ER*α* was expressed in the cells. ER*α* was detected as a red color. **(D)** Evaluation of co-localization carried out by co-staining for FLAG-ER*α*-NTD(AF-1) and full-length ER*α*. After exclusive detection of FLAG-ER*α*-NTD(AF-1) (green, panel **Da**) and full-length ER*α* (red, panel **Db**), merged immunolabeling was observed (yellow, panel **Dc**).

First, when neither FLAG-ER*α*-NTD(AF-1) nor ER*α* was transfected into the HeLa cells, it was confirmed that the primary antibodies used—namely, anti-FLAG polyclonal antibody and anti-ER*α* monoclonal antibody—did not show any background staining (Fig 5A). FLAG-ER*α*-NTD(AF-1) peptide was detected only in the cytoplasm (Fig 5B) when FLAG-ER*α*-NTD(AF-1) was solely transfected and expressed in the largest transfection amount (0.5 *μ*g/well, where “well” refers to a well of the Imaging Chamber CG 8 Well used for the cell culture for image analysis). This introductory amount of FLAG-ER*α*-NTD(AF-1) is equivalent to the amount necessary for the full inhibition of ER*α*. There was absolutely no staining at the cell nuclei, only exclusive staining at the cytoplasm (Fig 5B). Such cytoplasmic localization, i.e., occurrence only in the cytoplasm, of FLAG-ER*α*-NTD(AF-1) was retained throughout the experiment (more than 3 days). By contrast, when full-length ER*α* was solely transfected, its immunostaining was observed exclusively in the cell nucleus from the very start of process (Fig 5C).

Full-length ER*α* includes the nuclear localization signal (NLS) at the region named *hinge*, which lies between the DBD and the LBD (Fig 1A) [23, 24]. It should be noted, however, that ER*α*-NTD(AF-1) does not have such an NLS or any NLS-like signals. If FLAG-ER*α*-NTD(AF-1) was expressed simultaneously with full-length ER*α* in the same cell preparation, full-length ER*α* would transfer this synthetic FLAG-ER*α*-NTD(AF-1) from the cytoplasm into the cell nucleus, forming an ER*α*-NTD(AF-1)•ER*α* complex. Since co-expressed synthetic ER*α*-NTD(AF-1) is tagged with the N-terminal FLAG-epitope peptide, their nuclear translocation would be monitored immunocytochemically using each specific antibody. Conversely, this translocation becomes a demonstration of the intermolecular interaction between FLAG-ER*α*-NTD(AF-1) and full-length ER*α*. Such actual experimental proof was indeed realized for the untethered free ER*α*-NTD(AF-1) fragment in this study.

Fig 5Da shows the anti-FLAG immunostaining of FLAG-ER*α*-NTD(AF-1) in the HeLa cells when co-expressed together with full-length ER*α*. Alexa Fluor 488’s green immunolabeling for FLAG-ER*α*-NTD(AF-1) was observed only in the cell nucleus and not in the cytoplasm. In spite of this green immunostaining, the same nucleus was stained red by the observation at 550 nm (Fig 5Db). This is because of the Cy3 immunolabeling of full-length ER*α*. As a result, as shown in Fig 5Dc, merging of the green and red images yielded an entirely yellow immunolabeling image. These observations confirmed that the full-length ER*α* and FLAG-ER*α*-NTD(AF-1) peptide were completely co-localized in the cell nucleus, clearly revealing that separately expressed untethered free FLAG-ER*α*-NTD(AF-1) interacts and combines with full-length ER*α* to move from the cytoplasm into the nucleus.

## Discussion

### Conformation of ER*α*-NTD(AF-1) for interaction with LBD(AF-2)

In the present study, two distinct experimental results indicated that the N-terminal domain of ER*α*, namely, ER*α*-NTD(AF-1), interacts directly with the C-terminal ligand-binding domain ER*α*-LBD(AF-2). These results are: (i) separately expressed untethered free ER*α*-NTD(AF-1) inhibited full-length ER*α* without competing with the agonist E2; and (ii) separately expressed untethered free ER*α*-NTD(AF-1) was translocated from the cytoplasm into the cell nucleus when co-expressed with full-length ER*α*. These findings were obtained based on the discovery by Yi et al. [5] that ER*α*-NTD(AF-1) is adjacent to LBD(AF-2) in the ER*α*•DNA complex and on our subsequent speculation that NTD(AF-1) and LBD(AF-2) must interact directly with each other. Since ER*β*-NTD(AF-1) did not inhibit full-length ER*α*, at least in terms of estrogen receptors, this NTD(AF-1) and LBD(AF-2) are specific to the ER*α* receptor.

The question of which part of NTD(AF-1) is adjacent to LBD(AF-2) is not answered in the literature [5]. Our structural analysis suggested the loop-containing region(s) of H3–H4 and/or H8–H9 of ER*α*-LBD(AF-2) as a site or sites to which NTD(AF-1) binds (Fig 1B). In general, the structure of NTD(AF-1) in various members of the nuclear receptor superfamily is characterized by a conformation lacking a stable three-dimensional (3D) structure and having very high flexibility [25–27]. The structure of ER*α*-NTD(AF-1) was also reported to be flexible and disordered [28, 29].

**Fig 6:**
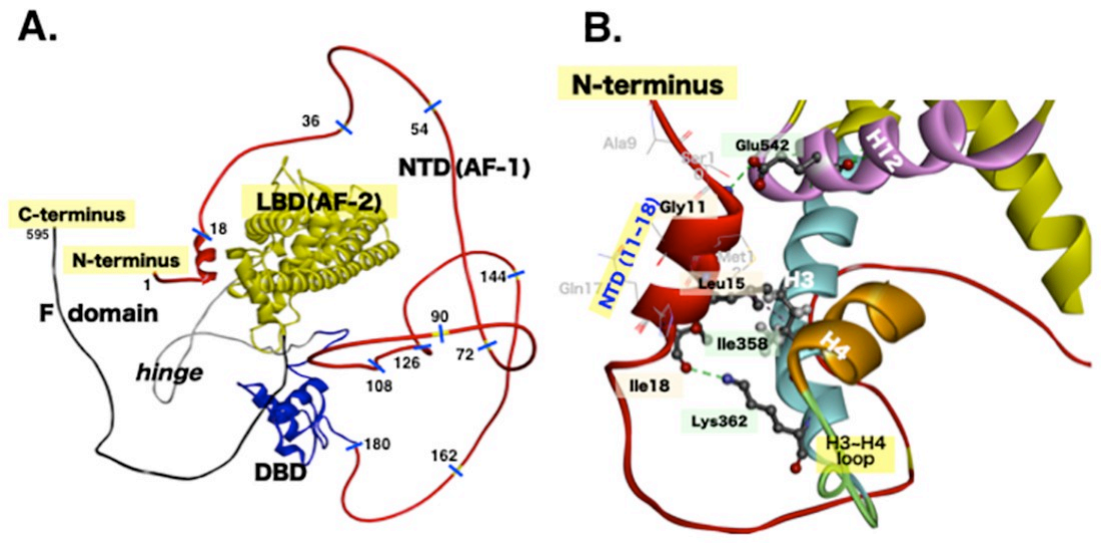
AlphaFold structural prediction of human estrogen receptor ER*α*. **(A)** Predicted 3D structure of the full-length ER*α* protein (1–595). This AlphaFold structure prediction is available on the AlphaFold website: https://alphafold.ebi.ac.uk/entry/P03372. P03372, a predicted 3D structure of full-length ER*α*, was further analyzed in detail using the software package DiscoveryStudio. The designated domain structures are the NTD(AF-1), DBD, *hinge*, LBD, and F domain, arranged from the N-terminus to the C-terminus. **(B)** Structural details of the domain‒domain interaction between the N-terminal *α*-helix (11–18) in ER*α*-NTD(AF-1) and the H3-loop-H4 region adjacent to H12/AF-2 in ER*α*-LBD.

It has recently become feasible to predict the 3D structures of proteins with considerably high accuracy by using a deep-learning artificial intelligence (AI) system. In particular, the AI system AlphaFold can depict the 3D structure of a protein from its amino acid sequence [30, 31]. According to the AlphaFold website, “The AlphaFold data base provides open access to over 200 million protein structure predictions to accelerate scientific research” [32]. When we used this database to determine and analyze the 3D structure of full-length ER*α*, AlphaFold presented one structure (P03372) from 429 structures in the protein data bank (PDB) [33]. We further analyzed the predicted structure in detail using the software package DiscoveryStudio. As reported in PDB, the DNA-binding domain (DBD: 181–263) and ligand-binding domain (LBD: 303–552) possess the best-known ordered 3D structures (Fig 6A). Most of the other domains [NTD(AF-1), *hinge*, and F-domain] have intrinsically disordered structures, i.e., they lack *α*-helices and *β*-strands.

The only exception is the *α*-helix in NTD(AF-1). At positions 11–18, the secondary structure *α*-helix was found to lie in the two helical turns (Fig 6A and 6B). In addition, this short *α*-helix was found to interact with ER*α*-LBD(AF-2) (Fig 6B). There are two hydrogen bonds, namely, Gly11-*α*NH⋯H12 Glu542-*δ*CO (1.79 Å) and Ile18-*α*CO⋯H3 Lys362-*ε*NH (2.59 Å), and three hydrophobic bonds, namely, Leu14⋯H3 Ile358 (5.11 Å), Leu14⋯H12 Leu539 (3.27 Å), and Leu15⋯H3 Ile358 (5.32 Å). These bonds appear to confirm a solid interaction between the short *α*-helix in NTD(AF-1) and the C-terminal LBD(AF-2). As a consequence, the short *α*-helix appears to interact with several amino acid residues in H3 and H12 present in ER*α*-LBD(AF-2).

It should be noted that the H3-loop-H4 region on the LBD surface area is adjacent to the H12/AF-2 site in ER*α* (Fig 1B). It is highly likely that the separately expressed untethered free ER*α*-NTD(AF-1) peptide is in a conformation similar to that of NTD(AF-1) in full-length ER*α*. In addition to the N-terminal *α*-helix (11–18) of intact tethered ER*α*-NTD(AF-1), the untethered free ER*α*-NTD(AF-1) peptide would also possess an *α*-helix (11–18) to interact with the H3-loop-H4 and LBD H12/AF-2 region. This implies that untethered free ER*α*-NTD(AF-1) peptide and the intact tethered ER*α*-NTD(AF-1) compete to interact with these regions. If the interaction between the untethered free ER*α*-NTD(AF-1) peptide and the H3-loop-H4 and LBD H12/AF-2 region fails to establish a proper conformation of the binding site for the coactivator proteins, such interaction competition must result in the inhibition of the transcription activation activity of intact full-length ER*α*. It is highly likely that, when NTD(AF-1) is properly linked to the central DBD domain, the *α*-helix structural unit at positions 11–18 plays a crucial role in ER*α*’s recruitment of the SRC coactivator proteins.

### Nuclear translocation of ER*α*-NTD(AF-1) by interacting with LBD(AF-2)

**Fig 7:**
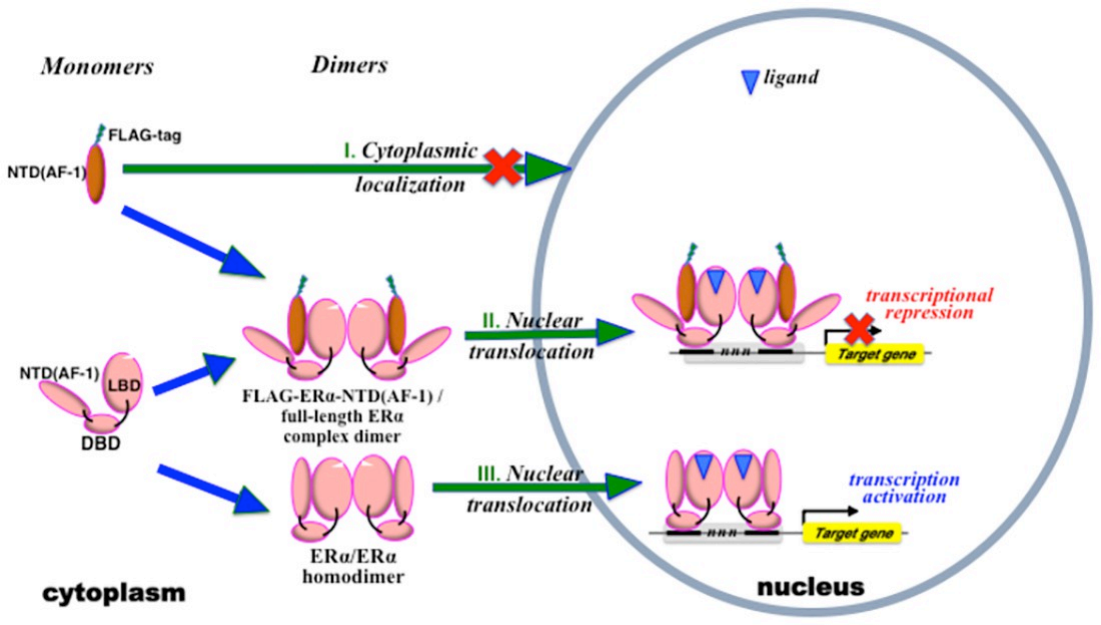
Outline flowchart of the nuclear translocation of ER*α*-NTD(AF-1)/ER*α* complex and transcription inhibition resulting from complexation in the cell. The figure shows only the mechanisms for FLAG-tagged ER*α*-NTD(AF-1), but exactly the same mechanisms are valid for non-tagged ER*α*-NTD(AF-1). Expressed FLAG-ER*α*-NTD(AF-1) has no nuclear localization signal (NLS), and therefore it remains in the cytoplasm (case I). When co-expressed together with full-length ER*α*, the resulting FLAG-ER*α*-NTD(AF-1)•ER*α* complex migrates from the cytoplasm into the nucleus (case II). This complex is not active in the luciferase reporter gene assay. ER*α* dimerized in the cytoplasm moves into the nucleus (case III).

In Fig 7, a putative molecular mechanism of nuclear translocation of untethered free ER*α*-NTD(AF-1) is depicted together with its mechanism of inhibitory activity against full-length ER*α*. The cytoplasmic localization is characteristic of the 1–180 peptide fragment of ER*α*, specifically untethered free ER*α*-NTD(AF-1), when expressed alone (case I) (Fig 7). This was demonstrated by an immunocytochemical staining study using FLAG-tagged ER*α*-NTD(AF-1) (Fig 5). ER*α*-NTD(AF-1) does not have an NLS signal, unlike full-length ER*α*, which can be translocated from the cytoplasm into the nucleus due to the signal (case III) (Fig 7). Straddling the C-terminal LBD(AF-2) of full-length ER*α*, untethered free ER*α*-NTD(AF-1) or its FLAG-tagged ER*α*-NTD(AF-1) peptide can move into the nucleus despite the absence of the NLS signal (case II) (Fig 7).

Untethered free ER*α*-NTD(AF-1) peptide forms an ER*α*-NTD(AF-1)•ER*α* complex by binding to the region involving H3-loop-H4 and H12/AF-2 in ER*α*-LBD (Fig 6B). It is unclear whether the interaction mode of untethered free ER*α*-NTD(AF-1) peptide with ER*α*-LBD(AF-2) is exactly the same as that of intact tethered ER*α*-NTD(AF-1) with its own ER*α*-LBD(AF-2). However, this interaction certainly resulted in the inhibition of the transcription activity of full-length ER*α*.

The complexation between the untethered free ER*α*-NTD(AF-1) peptide and the full-length ER*α* protein would enable the translocation of untethered free ER*α*-NTD(AF-1) into the nucleus: that is, full-length ER*α* transports ER*α*-NTD(AF-1) from the cytoplasm into the nucleus (case II) (Fig 7). Exactly the same nuclear translocation of ER*α*-NTD(AF-1) was brought about by the truncated ER*α*-derivative desNTD(AF-1)-ER*α* (data not shown).

Full-length ER*α* exists as a dimer via the specific dimerization that first occurs at the H10 region of LBD(AF-2) (Fig 1B). As a result, there are two types of ER*α*-NTD(AF-1)•ER*α* complex: one with complexation at both of the LBD(AF-2) domains as shown in case II (Fig 7) and one with complexation at only one of the two LBD(AF-2) domains in a dimer (not shown). In both cases, ER*α*-NTD(AF-1) is translocated into the nucleus.

### Inhibitory mode of untethered free ER*α*-NTD(AF-1)

It should be noted that the ER*α*-NTD(AF-1)•ER*α* complexes possess a sound, undamaged DNA-binding domain DBD in the full-length ER*α* protein, and therefore complexes carrying untethered free ER*α*-NTD(AF-1) can bind to DNA through conserved estrogen response element sequence motifs. However, as demonstrated in this study, the formed DNA complexes fail to properly recruit the coactivator protein SRC, likely because of the presence of “additive” untethered free ER*α*-NTD(AF-1) peptide. A physiologically inactive ER*α*-NTD(AF-1)•ER*α* complex has two NTD(AF-1) moieties: a co-expressed untethered free NTD(AF-1) peptide and an intact ER*α*-tethered NTD(AF-1) moiety. The challenge lies in elucidating why intact NTD(AF-1) functions as a stimulator while untethered free NTD(AF-1) functions as an inhibitor.

Even if untethered free ER*α*-NTD(AF-1) peptide is able to construct a binding site for the SRC coactivator, the intact ER*α*-NTD(AF-1) domain, a larger protein unit having 180 amino acid residues, might obstruct or impede the binding of SRC protein due to its steric hindrance. Perhaps the intact ER*α*-NTD(AF-1) plays some roles in preventing the acceptance of coactivator proteins. On the other hand, since only a small part of the co-expressed untethered free NTD(AF-1) peptide, namely, the *α*-helix (11–18), appears to bind to ER*α*-LBD (Fig 6B), the large remaining disordered part may also play a role in such hindrance or interference.

### Inquiry into the intrinsic functional role of ER*α*-NTD(AF-1)

DesNTD(AF-1)-ER*α* is almost equivalent to the native ER*α* isoform named ER*α*46, which was reported to be a weak transcriptional activator compared to ER*α* [34, 35]. ER*α*46 is 4 amino acids longer than desNTD(AF-1)-ER*α* at its N-terminus. As an E2-inducible activator, ER*α*46 has been reported to be 20–100% as efficient as full-length ER*α* in assays using various cells and promoter reporter genes. Recently, ER*α*46 was reported to be involved in the growth and metastasis of breast cancer cells [36]. Thus, what is its physiological role in breast cancer cells?

In this study, desNTD(AF-1)-ER*α* exhibited ca. 65% activity of full-length ER*α*. This result implies that ER*α* does not necessarily require NTD(AF-1) for its transcription activity. ER*α*46 shares an identical DNA sequence with wild-type full-length ER*α* from positions 174 to 595 [37, 38], and thus they share only AF-2 of the two activation function motifs. In the transcription activation mechanism, however, it is necessary for both ER*α*46 and full-length ER*α* to bind to the co-activator SRC and additionally to the secondary co-activator, p300. To recruit these co-activator proteins, full-length ER*α* utilizes both AF-1 and AF-2. ER*α*46 possesses only AF-2, and thus it has to utilize its LBD(AF-2) domain exclusively to recruit an SRC co-activator protein.

On the recruitment of the p160 coactivator proteins, SRCs utilize a region known as the “nuclear receptor interaction domain” (NID). NID contains three helical motifs sharing an LXXLL core consensus sequence, referred to as the nuclear receptor NR boxes I, II, and III. Research suggests that distinct NR boxes interact differently with various nuclear receptors, indicating that a single NR box is sufficient for activation by the estrogen receptor ER*α* [39]. This was verified for the SRC-1 and SRC-2 NR boxes; ER*α* showed a distinct preference for NR box II [40]. In contrast, SRC-3 utilizes both NR boxes I and II simultaneously for ER*α*, although ER*α* exhibits a bigger preference (approximately 2-fold) for NR box I than for box II [40], In this scenario, it is likely that NTD(AF-1) and LBD(AF-2) cooperate to form a specific binding site for the recruitment of these SRC proteins.

### Perspectives on the structural role of N-terminal NTD(AF-1) domain

Because of the lack of NTD(AF-1), ER*α*46 should recruit SRC proteins differently from full-length ER*α*. In this case, ER*α*46 uses its LBD(AF-2) for recruitment. It should be noted that, when ER*α*46 is co-expressed with full-length ER*α*, ER*α*46 inhibits ER*α* exclusively by forming their heterodimer [41, 42]. In the ER*α*46•ER*α* heterodimer, ER*α*46 lacks the adjacent NTD(AF-1) domain in its C-terminal LBD(AF-2), whereas the partner ER*α* has NTD(AF-1) and LBD(AF-2) naturally adjacent to each other. Consequently, the heterodimer ER*α*46•ER*α* possesses non-equivalent SRC-binding sites, recruiting the SRC coactivator for each. However, these coactivators probably exhibit spatial asymmetry, leading to a failure to recruit the secondary coactivator p300 or to form a transcriptionally productive p160•p300 complex.

Another ER*α* isoform, named ER*α*-LBD, has recently been identified in MCF-7 cells and reportedly promotes breast cancer and endocrine resistance [43]. This ER*α*-LBD isomer would form also a heterodimer with full-length ER*α*, and the resulting ER*α*-LBD•ER*α* heterodimer would be nonproductive.

Formation of ER*α* heterodimers such as ER*α*46•ER*α* and ER*α*-LBD•ER*α* might hinder the turnover of intact full-length ER*α*, impacting or promoting the proliferation of cancer cells. Thus, ER*α*-NTD(AF-1) appears to be a molecular determinant of the cellular state: its presence in the full-length natural estrogen receptor ER*α* determines its physiological role as a nuclear receptor, while its absence in the isomer ER*α*46 or ER*α*-LBD might determine its pathophysiological role as a molecular carcinogenic or tumor progressive factor.

The structural analysis of the ER*α*-coactivator complex on DNA suggests the N/C-intramolecular interaction between the N-terminal ER*α*-NTD(AF-1) and the C-terminal ER*α*-LBD(AF-2). We then demonstrated in the present study the intermolecular interaction between the untethered free ER*α*-NTD(AF-1) and the LBD(AF-2) domain of full-length ER*α*. Since this is the indirect evidence, however, it is now crucial to prove the interaction directly using biochemical methods. We are preparing to conduct an experiment to provide such evidence.

## Conclusion

Based on structural information about the DNA-bound ER*α*-coactivator complex, we revealed that, to construct the binding sites of coactivator SRC proteins, ER*α*-NTD(AF-1) interacts with the region including the H3-loop-H4 and H12/AF-2 in the C-terminal ER*α*-LBD(AF-2) domain. This N/C-intramolecular interaction was demonstrated by two pieces of evidence: untethered free ER*α*-NTD(AF-1) inhibited the transcription activity of full-length ER*α* in the luciferase reporter gene assay, and the cytoplasm-to-nucleus translocation of ER*α*-NTD(AF-1) was immunocytochemically detected only when co-expressed with full-length ER*α*. The present results offer insights into the molecular design of ER*α*-specific inhibitors, independent of receptor antagonist.

## Abbreviations

3D: three-dimensional
AF: activation function
AI: artificial intelligence
cryo-EM: cryoelectron microscopy
DBD: DNA-binding domain
desNTD(AF-1)-ER*α*: ER*α* C-terminal fragment with amino acid residues 181–595
E2: 17*β*-estradiol
ER*α*-NTD(AF-1): ER*α* N-terminal fragment with amino acid residues 1–180
ER*α*: estrogen receptor alpha
ERE: estrogen response element
H12: *α*-helix No. 12
LBD: ligand-binding domain
LBP: ligand-binding pocket
NID: nuclear receptor interaction domain
NLS: nuclear localization signal
NR: nuclear receptor
NTD: N-terminal domain
PBS: phosphate buffered saline
PDB: protein data bank
SRC: steroid receptor coactivator

## References

[1] Arnal JF, Lenfant F, Metivier R, Flouriot G, Henrion D, Adlanmerini M, et al. Membrane and nuclear estrogen receptor alpha actions: from tissue specificity to medical implications. Physiol Rev. 2017;97(3): 1045–1087. doi: 10.1152/physrev.00024.2016 28539435

[2] Fuentes N, Silveyra P. Estrogen receptor signaling mechanisms. Adv Protein Chem Struct Biol. 2019;116, 135–170. doi: 10.1016/bs.apcsb.2019.01.001 31036290 PMC6533072

[3] Liu Y, Ma H, Yao J. ER*α*, A key target for cancer therapy: a review. Onco Targets Ther. 2020;13: 2183–2191. doi: 10.2147/OTT.S236532 32210584 PMC7073439

[4] Clusan L, Ferrière F, Flouriot G, Pakdel F. A basic review on estrogen receptor signaling pathways in breast cancer. Int J Mol Sci. 2023;24(7): 6834. doi: 10.3390/ijms24076834 37047814 PMC10095386

[5] Yi P, Wang Z, Feng Q, Pintilie GD, Foulds CE, Lanz RB, et al. Structure of a biologically active estrogen receptor-coactivator complex on DNA. Mol Cell 2015;57(6): 1047–1058. doi: 10.1016/j.molcel.2015.01.025 25728767 PMC4369429

[6] Shiau AK, Barstad D, Radek JT, Meyers MJ, Nettles KW, Katzenellenbogen BS, et al. Structural characterization of a subtype-selective ligand reveals a novel mode of estrogen receptor antagonism. Nat Struct Biol. 2002;9(5): 359–364. 11953755 10.1038/nsb787

[7] Darimont BD, Wagner RL, Apriletti JW, Stallcup MR, Kushner PJ, Baxter JD, et al. Structure and specificity of nuclear receptor-coactivator interactions. Genes Dev. 1998;12(21): 3343–3356. doi: 10.1101/gad.12.21.3343 9808622 PMC317236

[8] Nolte RT, Wisely GB, Westin S, Cobb JE, Lambert MH, Kurokawa R, et al. Ligand binding and co-activator assembly of the peroxisome proliferator-activated receptor-gamma. Nature. 1998;395(6698): 137–143. doi: 10.1038/25931 9744270

[9] York B, O’Malley BW. Steroid receptor coactivator (SRC) family: masters of systems biology. J Biol Chem. 2010;285(50): 38743–38750. doi: 10.1074/jbc.R110.193367 20956538 PMC2998129

[10] Stashi E, York B, O’Malley BW. Steroid receptor coactivators: servants and masters for control of systems metabolism. Trends Endocrinol Metab. 2014;25(7): 337–347. doi: 10.1016/j.tem.2014.05.004 24953190 PMC4108168

[11] Shiau AK, Barstad D, Loria PM, Cheng L, Kushner PJ, Agard DA, et al. The structural basis of estrogen receptor/coactivator recognition and the antagonism of this interaction by tamoxifen. PLoS Genet. 2011 Oct;7(10):e1002337. 9875847 10.1016/s0092-8674(00)81717-1

[12] Matsushima A, Liu X, Okada H, Shimohigashi M, Shimohigashi Y. Bisphenol AF is a full agonist for the estrogen receptor ER*α* but a highly specific antagonist for ER*β*. Environ Health Perspect. 2010;118(9): 1267–1272. doi: 10.1289/ehp.0901819 20427257 PMC2944088

[13] Liu X, Suyama K, Shiki J, Torikai K, Nose T, Shimohigashi M, et al. Bisphenol AF: Halogen bonding effect is a major driving force for the dual ER*α*-agonist and ER*β*-antagonist activities. Bioorg Med Chem. 2020;28(3): 115274. doi: 10.1016/j.bmc.2019.115274 31879182

[14] Hopp TP, Prickett KS, Price VL, Libby RT, March CJ, Pat Cerretti D, et al. A short polypeptide marker sequence useful for recombinant protein identification and purification. Nat Biotechnol. 1988;6: 1204–1210. https://www.nature.com/articles/nbt1088-1204

[15] Liu X, Matsushima A, Nakamura M, Costa T, Nose T, Shimohigashi Y. Fine spatial assembly for construction of the phenol-binding pocket to capture bisphenol A in the human nuclear receptor estrogen-related receptor *γ*. J Biochem. 2012;151(4): 403–415. doi: 10.1093/jb/mvs008 22298789

[16] Liu X, Matsushima A, Shimohigashi M, Shimohigashi Y. A characteristic back support structure in the bisphenol A-binding pocket in the human nuclear receptor ERR*γ*. PLoS One. 2014;9(6): e101252. doi: 10.1371/journal.pone.0101252 24978476 PMC4076284

[17] Liu X, Matsuyama Y, Shimohigashi M, Shimohigashi Y. ER*α*-agonist and ER*β*-antagonist bifunctional next-generation bisphenols with no halogens: BPAP, BPB, and BPZ. Toxicol Lett. 2021;345: 24–33. doi: 10.1016/j.toxlet.2021.04.001 33857583

[18] Liu X, Suyama K, Nose T, Shimohigashi M, Shimohigashi Y. Bisphenol-C is the strongest bifunctional ER*α*-agonist and ER*β*-antagonist due to magnified halogen bonding. PLoS One 2021;16(2): e0246583. doi: 10.1371/journal.pone.0246583 33561155 PMC7872235

[19] Matthews JC, Hori K, Cormier MJ. Purification and properties of Renilla reniformis luciferase. Biochemistry 1977;16(1): 85–91. doi: 10.1021/bi00620a014 12797

[20] Liu X, Nishimura H, Fujiyama A, Matsushima A, Shimohigashi M, Shimohigashi Y. *α*-Helix-peptides comprising the human nuclear receptor ERR*γ* competitively provoke inhibition of functional homomeric dimerization. Biopolymers. 2016;106(4): 547–554. doi: 10.1002/bip.22795 26662629

[21] Arunklakshana O, Schild HO. Some quantitative uses of drug antagonists. Br J Pharmacol Chemother. 1959;14(1): 48–58. doi: 10.1111/j.1476-5381.1959.tb00928.x 13651579 PMC1481829

[22] Ghosh K, Kowal D, Dawson LA, Tasse R. Design and models for estimating antagonist potency (pA_2_, K_d_ and IC_50_) following the detection of antagonism observed in the presence of intrinsic activity. Neuropharmacology. 1999;38(3): 361–373. doi: 10.1016/S0028-3908(98)00185-3 10219974

[23] Casa AJ, Hochbaum D, Sreekumar S, Oesterreich S, Lee AV. The estrogen receptor alpha nuclear localization sequence is critical for fulvestrant-induced degradation of the receptor. Mol Cell Endocrinol. 2015;415: 76–86. doi: 10.1016/j.mce.2015.08.007 26272024

[24] Yaşar P, Ayaz G, User SD, Güpür G, Muyan M. Molecular mechanism of estrogen-estrogen receptor signaling. Reprod Med Biol. 2016;16(1): 4–20. doi: 10.1002/rmb2.12006 29259445 PMC5715874

[25] Kumar R, Thompson EB. Folding of the glucocorticoid receptor N-terminal transactivation function: dynamics and regulation. Mol Cell Endocrinol. 2012;348(2): 450–456. doi: 10.1016/j.mce.2011.03.024 21501657

[26] Li J, Motlagh HN, Chakuroff C, Thompson EB, Hilser VJ. Thermodynamic dissection of the intrinsically disordered N-terminal domain of human glucocorticoid recepto. J Biol Chem. 2012;287(32): 26777–26787. doi: 10.1074/jbc.M112.355651 22669939 PMC3411015

[27] Kumar R, Moure CM, Khan SH, Callaway C, Grimm SL, Goswami D, et al. Regulation of the structurally dynamic N-terminal domain of progesterone receptor by protein-induced folding. J Biol Chem. 2013;288(42): 30285–30299. doi: 10.1074/jbc.M113.491787 23995840 PMC3798494

[28] Wärnmark A, Wikström A, Wright AP, Gustafsson JA, Härd T. The N-terminal regions of estrogen receptor *α* and *β* are unstructured in vitro and show different TBP binding properties. J Biol Chem. 2001;276(49): 45939–45944. doi: 10.1074/jbc.M107875200 11595744

[29] Rajbhandari P, Finn G, Solodin NM, Singarapu KK, Sahu SC, Markley JL, et al. Regulation of estrogen receptor *α* N-terminus conformation and function by peptidyl prolyl isomerase Pin1. Mol Cell Biol. 2012;32(2): 445–457. doi: 10.1128/MCB.06073-11 22064478 PMC3255769

[30] Jumper J, Evans R, Pritzel A, Green T, Figurnov M, Ronneberger O, et al. Highly accurate protein structure prediction with AlphaFold. Nature. 2021;596(7873): 583–589. doi: 10.1038/s41586-021-03819-2 34265844 PMC8371605

[31] Varadi M, Anyango S, Deshpande M, Nair S, Natassia C, Yordanova G, et al. AlphaFold Protein Structure Database: massively expanding the structural coverage of protein-sequence space with high-accuracy models. Nucleic Acids Res. 2022;50(D1): 820 D439–D444. doi: 10.1093/nar/gkab1061 34791371 PMC8728224

[32] AlphaFold protein structure database. https://alphafold.com/.

[33] Estrogen receptor: AlphaFold structure prediction. https://alphafold.ebi.ac.uk/entry/P03372.

[34] Berry M, Metzger D, Chambon P. Role of the two activating domains of the oestrogen receptor in the cell-type and promoter-context dependent agonistic activity of the anti-oestrogen 4-hydroxytamoxifen. EMBO J. 1990;9(9): 2811–2818. doi: 10.1002/j.1460-2075.1990.tb07469.x 2118104 PMC551992

[35] Figtree GA, McDonald D, Watkins H, Channon KM. Truncated estrogen receptor *α* 46-kDa isoform in human endothelial cells: relationship to acute activation of nitric oxide synthase. Circulation 2003:107(7): 120–126. doi: 10.1161/01.CIR.0000043805.11780.F5 12515753

[36] Cirillo F, Pellegrino M, Talia M, Perrotta ID, Rigiracciolo DC, Spinelli A, et al. Estrogen receptor variant ER*α*46 and insulin receptor drive in primary breast cancer cells growth effects and interleukin 11 induction prompting the motility of cancer-associated fibroblasts. Clin Transl Med. 2021;11(11): e516. doi: 10.1002/ctm2.516 34841688 PMC8567034

[37] Flouriot G, Brand H, Denger S, Metivier R, Kos M, Reid G, et al. Identification of a new isoform of the human estrogen receptor-alpha (hER-*α*) that is encoded by distinct transcripts and that is able to repress hER-*α* activation function 1. EMBO J. 2000;19(17): 4688–4700. doi: 10.1093/emboj/19.17.4688 10970861 PMC302047

[38] Saito K, Cui H. Estrogen receptor alpha splice variants, post-translational modifications, and their physiological functions. Cells 2023;12(6): 895. doi: 10.3390/cells12060895 36980236 PMC10047206

[39] McInerney EM, Rose DW, Flynn SE, Westin S, Mullen TM, Krones A, et al. Determinants of coactivator LXXLL motif specificity in nuclear receptor transcriptional activation. Genes Dev. 1998;12(21): 3357–3368. doi: 10.1101/gad.12.21.3357 9808623 PMC317227

[40] Bramlett KS, Wu Y, Burris TP. Ligands specify coactivator nuclear receptor (NR) box affinity for estrogen receptor subtypesl. Mol Endocrinol. 2001;15(6): 909–922. doi: 10.1210/mend.15.6.0649 11376110

[41] Kampa M, Pelekanou V, Notas G, Stathopoulos EN, Castanas E. The estrogen receptor: two or more molecules, multiple variants, diverse localizations, signaling and functions. Are we undergoing a paradigm-shift as regards their significance in breast cancer?. Hormones (Athens) 2013;12(1): 69–85. doi: 10.1007/BF03401288 23624133

[42] Chantalat E, Boudou F, Laurell H, Palierne G, Houtman R, Melchers D, et al. The AF-1-deficient estrogen receptor ER*α*46 isoform is frequently expressed in human breast tumors. Breast Cancer Res. 2016;18(1): 123. doi: 10.1186/s13058-016-0780-7 27927249 PMC5142410

[43] Strillacci A, Sansone P, Rajasekhar VK, Turkekul M, Boyko V, Meng F, et al. ER*α*-LBD, an isoform of estrogen receptor alpha, promotes breast cancer proliferation and endocrine resistance. NPJ Breast Cancer. 2022;8(1): 96. doi: 10.1038/s41523-022-00470-6 35999225 PMC9399095

