# Supplementary material for: The N-terminal activation function AF-1 domain of ERα interacts directly with the C-terminal AF-2-holding ligand-binding domain to recruit the coactivator proteins
Source: PLoS One. 2024 Oct 21;19(10):e0312276. doi: 10.1371/journal.pone.0312276 (PMC11493271; doi:10.1371/journal.pone.0312276)
Supplement: Attachment — Submitted filename: Response to Reviewers.pdf [file pone.0312276.s001.pdf]

Responses to Reviewers

August 10, 2024

Re.: **PONE-D-24-22407**

Title:
The N-terminal activation function AF-1 domain of ERα interacts directly with the C-terminal AF-2-holding ligand-binding domain to recruit the coactivator proteins

Authors:
Xiaohui Liu, Yutaka Matsuyama, Makiko Sugiyama, Keitaro Suyama, Takeru Nose, Miki Shimohigashi, and Yasuyuki Shimohigashi

Index

Titles	Page
I. Responses to the academic editor	2
II. Responses to Journal requirements	3
III. Responses to Reviewer #1	8

I. Responses to the academic editor

Blue: Comments and suggestions from academic editor.

Black: Our answer to the academic editor.

❖ Please include the following items when submitting your revised manuscript:

- A rebuttal letter that responds to each point raised by the academic editor and reviewer(s). You should upload this letter as a separate file labeled 'Response to Reviewers'.

Answer: Yes, we did upload the rebuttal letter in a separate file labeled 'Response to Reviewers'.

- A marked-up copy of your manuscript that highlights changes made to the original version. You should upload this as a separate file labeled 'Revised Manuscript with Track Changes'.

Answer: Yes, we did upload a marked-up copy of our manuscript. The changes made for original version was highlighted with the yellow marker for corrected words, sentences, or numbers in red color. These changes were carried out in a separate file labeled "Revised Manuscript with Track Changes".

- An unmarked version of your revised paper without tracked changes. You should upload this as a separate file labeled 'Manuscript'.

Answer: Yes, we did upload an unmarked version of our revised paper without tracked changes. The file was prepared in a separate file labeled "Manuscript".

❖ If you would like to make changes to your financial disclosure, please include your updated statement in your cover letter. Guidelines for resubmitting your figure files are available below the reviewer comments at the end of this letter.

Answer: No, we have no changes to our financial disclosure.

We have made our largest efforts to prepare the figure files in the PACE format according to your Guidelines.

❖ If applicable, we recommend that you deposit your **laboratory protocols** in protocols.io to enhance the reproducibility of your results. Protocols.io assigns your protocol its own identifier (DOI) so that it can be cited independently in the future. For instructions see: : https //journals.plos.org/plosone/s/submission-guidelines#loc-laboratory-protocols.

Additionally, PLOS ONE offers an option for publishing peer-reviewed Lab Protocol articles, which describe protocols hosted on protocols.io. Read more information on sharing protocols at https://plos.org/protocols?utmmedium=editorial-email&utm_source=authorletters&utm_campaign=protocols.

Answer: Thank you very much for valuable information about Lab Protocol. Unfortunately, we do not have any such Lab protocols at this moment.

II. Responses to Journal requirements

Blue: Journal requirements.

Black: Our answer to the Journal requirements.

Red: Sentences added newly in the old text.

When submitting your revision, we need you to address these additional requirements.

1. Please ensure that your manuscript meets PLOS ONE's style requirements, including those for file naming. The PLOS ONE style templates can be found at https://journals.plos.org/plosone/s/file?id=wjVg/PLOSOne_formatting_sample_main_body.pdf and https://journals.plos.org/plosone/s/file?id=ba62/PLOSOne_formatting_sample_title_authors_affiliations.pdf

Answer: Yes, we ensured that our manuscript meets PLOS ONE's style requirements, including those for file naming.

2. Please update your submission to use the PLOS LaTeX template. The template and more information on our requirements for LaTeX submissions can be found at http://journals.plos.org/plosone/s/latex.

Answer: Yes, we used the PLOS LaTeX template to prepare our final version of manuscript.

3 Thank you for stating the following financial disclosure:

[This work was supported by the Japan Society for the Promotion of Science (JSPS) KAKENHI; grant nos. 25740024, 15K00557, 19K12340 and 22K12395 (to X.L.) and 22221005 and 15H01741 (to Y.S.). This work was also supported in part by a Health and Labour Sciences Research Grant for Research on the Risk of Chemical Substances from the Ministry of Health, Labor, and Welfare of Japan, under the contract/grant no. H20-Chemistry-General-003 (to Y.S.).].

Please state what role the funders took in the study. If the funders had no role, please state: "The funders had no role in study design, data collection and analysis, decision to publish, or preparation of the manuscript."

If this statement is not correct you must amend it as needed.

Please include this amended Role of Funder statement in your cover letter; we will change the online submission form on your behalf.

Answer: Thank you very much for your kind advice about the amendment for Role of Funder statement. According to your indication, we newly added the statement "The funders had no role in study design, data collection and analysis, decision to publish, or preparation of the manuscript" in the Acknowledges section. Also, we included this amended Role of Funder statement in our cover letter to Academic Editor.

4. Please provide a complete Data Availability Statement in the submission form, ensuring you include all necessary access information or a reason for why you are unable to make your data freely accessible. If your research concerns only data provided within your submission, please write "All data are in the manuscript and/or supporting information files" as your Data Availability Statement.

Answer: **Yes,** we newly placed the section of Data Availability Statement in the revised manuscript, particularly in prior to the section of Acknowledges. According to your indication, we described, "All data are in the manuscript." in the statement. Because we do not have any supporting information files within our submission, we eliminated the phrase "and/or supporting information files". Is this all right?

5. We note that Figures 1, 6 and 7 in your submission contain [map/satellite] images which may be copyrighted. All PLOS content is published under the Creative Commons Attribution License (CC BY 4.0), which means that the manuscript, images, and Supporting Information files will be freely available online, and any third party is permitted to access, download, copy, distribute, and use these materials in any way, even commercially, with proper attribution. For these reasons, we cannot publish previously copyrighted maps or satellite images created using proprietary data, such as Google software (Google Maps, Street View, and Earth). For more information, see our copyright guidelines: http://journals.plos.org/plosone/s/licenses-and-copyright.

We require you to either (1) present written permission from the copyright holder to publish these figures specifically under the CC BY 4.0 license, or (2) remove the figures from your submission:

1. You may seek permission from the original copyright holder of Figures 1, 6 and 7 to publish the content specifically under the CC BY 4.0 license.

We recommend that you contact the original copyright holder with the Content Permission Form (http://journals.plos.org/plosone/s/file?id=7c09/content-permission-form.pdf) and the following text:
"I request permission for the open-access journal PLOS ONE to publish XXX under the Creative Commons Attribution License (CCAL) CC BY 4.0 (http://creativecommons.org/licenses/by/4.0/). Please be aware that this license allows unrestricted use and distribution, even commercially, by third parties. Please reply and provide explicit written permission to publish XXX under a CC BY license and complete the attached form."

Please upload the completed Content Permission Form or other proof of granted permissions as an ""Other"" file with your submission.

In the figure caption of the copyrighted figure, please include the following text: "Reprinted from [ref] under a CC BY license, with permission from [name of publisher], original copyright [original copyright year]."

2. If you are unable to obtain permission from the original copyright holder to publish these figures under the CC BY 4.0 license or if the copyright holder's requirements are incompatible with the CC BY 4.0 license, please either i) remove the figure or ii) supply a replacement figure that complies with the CC BY 4.0 license. Please check copyright information on all replacement figures and update the figure caption with source information. If applicable, please specify in the figure caption text when a figure is similar but not identical to the original image and is therefore for illustrative purposes only.

The following resources for replacing copyrighted map figures may be helpful:

USGS National Map Viewer (public domain): http://viewer.nationalmap.gov/viewer/
The Gateway to Astronaut Photography of Earth (public domain): http://eol.jsc.nasa.gov/sseop/clickmap/ Mapsat
the CIA (public domain): https://www.cia.gov/library/publications/the-world-factbook/index.html
and https://www.cia. gov/library/publications/cia-maps-publications/index.html
NASA Earth Observatory (public domain): http://earthobservatory.nasa.gov/
Landsat: http://landsat.visibleearth.nasa.gov/
USGS EROS (Earth Resources Observatory and Science (EROS) Center) (public domain): http://eros.usgs.gov/#
Natural Earth (public domain): http://www.naturalearthdata.com/

Answer: No, the Figures indicated do not contain any images copyrighted, although you wrote "We note that Figures 1, 6 and 7 in your submission contain [map/satellite] images which may be copyrighted". For more details, we explain here, respectively, as follows.

(1) Figure 1: Constructive features of human estrogen receptor α (ERα).

(i) Fig. 1A shows the bar charts of estrogen receptor α (ERα) fragment ERα-NTD(AF-1) and desNTD(AF-1)-ERα. These ERα fragments ERα-NTD(AF-1) and desNTD(AF-1)-ERα were designed and synthesized biochemically for the first time in this study, and thus **the original having a copyright does not exist for this figure**.

(ii) Fig. 1B shows the 3D schematic ribbon diagram representation of ERα-LBD in the C2 *symmetric structure*. As performed in the usual protein structural studies, the 3D structure of ERα-LBD, 1ERE (PDB ID code), was first downloaded from the Protein Data Base (PDB) into the software package DiscoveryStudio of our personal computer (PC). Fig. 1B was then produced as a screenshot showing the best depicture of ERα-LBD in the C2 symmetric structure. **Thus, the original having a copyright does not exist for this figure.** However, it is better to show the original PDB ID code of for this picturing, and then we newly added the following description in the figure legend of Fig. 1B. "The original 3D structure of this dimeric representation is 1G50 (PDB identification code), which was further analyzed using the software package DiscoveryStudio."

(iii) Fig. 1C shows a symbolized cartoon illustration of the structure of the ERα-coactivator-DNA complex. As indicated in the figure legend, an annotated cryo-EM density map and its illustration were reported by Yi *et al.* (2015) [5]. We prepared, by our own hands, a cartoon illustration of the structure of the ERα-coactivator-DNA complex to symbolize the complex structure. **Thus, the original having a copyright does not exist for this figure.**

Revised Figure Legend of Fig. 1

Fig. 1. Constructive features of human estrogen receptor α (ERα). (**A**) Schematic bar chart representation of domain structures A–F of ERα, ERα-NTD(AF-1), and desNTD(AF-1)-ERα. Each domain plays a different structural/functional role. Alias names representing domain functions are N-terminal domain, also known as activation function 1 (AF-1) [NTD(AF-1)]; DNA-binding domain (DBD); and ligand-binding domain (LBD). The *hinge* region between DBD and LBD is part of the flexible linker. (**B**) Ordinary 3D schematic ribbon diagram representation of ERα-LBD in the C2 *symmetric structure*. α-Helix No. 12 (H12) (red) functions as AF-2. The original 3D structure of this dimeric representation is 1G50 (PDB identification code), which was further analyzed using the software package DiscoveryStudio. (**C**) A symbolized cartoon illustration of the structure of the ERα-coactivator-DNA complex. For this complex, an annotated cryo-EM density map and its illustration were reported by Yi *et al.* (2015) [5]. SRC-3 is a p160 family transcriptional coactivator protein, and p300 is a secondary coactivator.

(2) Figure 6: AlphaFold structural prediction of human estrogen receptor ERα.

As well as Fig. 1., **for this Figure 6, the originals having a copyright do not exist.** The predicted 3D structure was downloaded from the AlphaFold website into the software package DiscoveryStudio of our PC to further analyze by us. As described in the text (lines 509-513 of the old version), "The AlphaFold data base provides open access to over 200 million protein structure predictions to accelerate scientific research [34]. When we used this database to determine and analyze the 3D structure of full-length ERα, AlphaFold presented one structure (P03372) from 429 structures in PDB [35]. "

In order to express such an analytical process in detail, we newly added the following description in the figure legend. "P03372, a predicted 3D structure of full-length ERα, was further analyzed in detail using the software package DiscoveryStudio."

(i) **Fig. 6A** shows the Predicted 3D structure of the full-length ERα protein (1–595). In this Fig. 6A, to provide a more plain figure, we processed the analysis further. For instance, the peptide chain was fragmented in the NTD. **Apparently, the original having a copyright does not exist for this figure.**

(ii) **Fig. 6B** shows the Structural details of the domain–domain interaction between the N-terminal α-helix (11–18) in ERα-NTD(AF-1) and the H3-loop-H4 region adjacent to H12/AF-2 in ERα-LBD. Figure depicts an expanded substructure showing intramolecular interactions between the N-terminal α-helix amino acid residues 11-18 and the residues of C-terminal H3-loop-H4/H12 region in ERα-LBD domain. We analyzed these interactions in detail also using the DiscoveryStudio software. **Thus, the original having a copyright does not exist also for this figure.**

Revised Figure Legend of Fig. 6

Fig. 6. AlphaFold structural prediction of human estrogen receptor ERα. (**A**) Predicted 3D structure of the full-length ERα protein (1–595). This AlphaFold structure prediction is available on the AlphaFold website: https://alphafold.ebi.ac.uk/entry/P03372. P03372, a predicted 3D structure of full-length ERα, was further analyzed in detail using the software package DiscoveryStudio. The designated domain structures are the NTD(AF-1), DBD, *hinge*, LBD, and F domain, arranged from the N-terminus to the C-terminus. (**B**) Structural details of the domain–domain interaction between the N-terminal α-helix (11–18) in ERα-NTD(AF-1) and the H3-loop-H4 region adjacent to H12/AF-2 in ERα-LBD.

(3) **Figure 7: Outline flowchart of the nuclear translocation of ERα-NTD(AF-1)/ERα complex and transcription inhibition resulting from complexation in the cell.**

For Fig. 7, the originals having a copyright do not exist. This figure is absolutely our original drawings based on our own ideas.

6. Please review your reference list to ensure that it is complete and correct. If you have cited papers that have been retracted, please include the rationale for doing so in the manuscript text, or remove these references and replace them with relevant current references. Any changes to the reference list should be mentioned in the rebuttal letter that accompanies your revised manuscript. If you need to cite a retracted article, indicate the article's retracted status in the References list and also include a citation and full reference for the retraction notice.

Answer: Yes, we have checked very carefully all the literatures in the References list, and ensured that the list was complete. There is no correction in all the literatures cited, and also there is no literature retracted.

III. Responses to Reviewer #1

Blue: Journal requirements.

Black: Our answer to the Journal requirements.

Red: Sentences added newly in the old text.

Reviewer's Responses to Questions
Comments to the Author

1. Is the manuscript technically sound, and do the data support the conclusions?

The manuscript must describe a technically sound piece of scientific research with data that supports the conclusions. Experiments must have been conducted rigorously, with appropriate controls, replication, and sample sizes. The conclusions must be drawn appropriately based on the data presented.
Reviewer #1: Yes

Answer: We greatly appreciate your appropriate response "Yes," an affirmative reply to the questions about evaluation of our study.

2. Has the statistical analysis been performed appropriately and rigorously?

Reviewer #1: I Don't Know

Answer: We have performed the statistical analysis appropriately and rigorously. This is definitely sure.

3. Have the authors made all data underlying the findings in their manuscript fully available?

The PLOS Data policy requires authors to make all data underlying the findings described in their manuscript fully available without restriction, with rare exception (please refer to the Data Availability Statement in the manuscript PDF file). The data should be provided as part of the manuscript or its supporting information, or deposited to a public repository. For example, in addition to summary statistics, the data points behind means, medians and variance measures should be available. If there are restrictions on publicly sharing data—e.g. participant privacy or use of data from a third party—those must be specified.

Reviewer #1: Yes

Answer: We greatly appreciate your appropriate response "Yes," an affirmative reply to the questions about the full availability of all data underlying the findings in our manuscript.

4. Is the manuscript presented in an intelligible fashion and written in standard English?

PLOS ONE does not copyedit accepted manuscripts, so the language in submitted articles must be clear, correct, and unambiguous. Any typographical or grammatical errors should be corrected at revision, so please note any specific errors here.

Reviewer #1: Yes

Answer: We greatly appreciate your appropriate response "Yes," an affirmative reply to the questions about the manuscript presentation whether it is in an intelligible fashion and written in Standard English.

Reviewer #1: REVIEW WITH MINOR REVISION COMMENT ADDED.

Liu et al have used a previously published cryoEM structure of human ERalpha, which shows that the N-terminal domain (NTD) and ligand binding domain (LBD) at the C-terminus are spatially close, to investigate the role in transcriptional activation of ERalpha of the interaction of N-terminal domain (NTD) with the ligand binding (LBD).

Their key finding is that the NTD from ERalpha can inhibit transcriptional activation of ERalpha. Interestingly, the NTD from ERbeta does not inhibit ERalpha. Thus, the inhibition of activation of ERalpha is specific to the NTD of ERalpha.

Then Liu et al provide a structural analysis of the interaction of the NTD from ERa with the full-length ERa using the cryoEM structure of ERalpha. This leads Liu et al to propose that binding of the ERalpha NTD to the LBD of ERalpha interferes with binding of SRC co-activator proteins, which in turn inhibits transcriptional activation of ERalpha. This is a reasonable hypothesis.

This is a very thorough and detailed study, which will be of much interest to endocrinologists studying ERalpha. The experiments are well-done. The data and figures are solid.

Answer: We greatly appreciated your very appropriate summary of our research results. We especially thank very much for the comment and judgment that "This is a very thorough and detailed study, which will be of much interest to endocrinologists studying ERalpha. The experiments are well-done. The data and figures are solid."

Reviewer #1: Minor Revision comments.
However, the writing needs editing from two perspectives.

First, in the Abstract: What is ERalpha-NTD(AF-1)? Is it the N-terminal domain [residues 1-180], without a DNA or ligand binding domain, or is it the full ERalpha [residues 1-595]? What is desNTD(AF-1)-ERa? Is it a truncated ERalpha [residues 181-595], containing the DBD, hinge and LBD?

I suggest adding explicit definitions with the residues to desNTD(AF-1)-ERa and ER-NTD(AF-1) to the Abbreviations Section.

Answer: We thank very much for your valuable comment about abbreviations of ERalpha fragments ERalpha-NTD(AF-1) and desNTD(AF-1)-ERalpha. ERalpha-NTD(AF-1) is ERalpha [residues 1–180], while desNTD(AF-1)-ERalpha is ERalpha [residues 181–595]. We decided to show these clearly in the Abstract.

Also, according to your courteous suggestion, we added these abbreviations to the Abbreviations Section.

Revision of the text in the manuscript (MS)

(i) in the **Abstract** (lines 47-49 of old version MS)

In the present study, we first evaluated untethered free ERα-NTD(AF-1) [residues 1–180] and its-truncated desNTD(AF-1)-ERα [residues 181–595] in a luciferase reporter gene assay.

(ii) in the **Abbreviations** (lines 66-71 of old version MS)

Abbreviations: AF, activation function; AI, artificial intelligence; cryo-EM, cryoelectron microscopy; DBD, DNA-binding domain; desNTD(AF-1)-ERα, ERα C-terminal fragment with amino acid residues 181–595; E2, 17β-estradiol; ERα, estrogen receptor alpha; ERα-NTD(AF-1), ERα N-terminal fragment with amino acid residues 1–180; ERE, estrogen response element; H12, α-helix No. 12; LBD, ligand-binding domain; LBP, ligand-binding pocket; NID, nuclear receptor interaction domain; NLS, nuclear localization signal; NR, nuclear receptor; NTD, N-terminal domain; PBS, phosphate buffered saline; PDB, protein data bank; SRC, steroid receptor coactivator; 3D, three-dimensional.

Reviewer #1: Further comments and suggestions from the reviewer

Also The text in line 279-285 on ERalpha46 could be condensed and moved to the end Discussion section. Keep the focus on the relevance for full-length ERalpha, which is the important biological estrogen receptor.

Answer: We thank very much for your appropriate suggestion about ERalpha46. We immediatedly recognized that the MS text line 279–285 is not suitable to place in the Results section, and that it would be much better to move to the Discussion section. Therefore, we removed the text in line 279–285, and then combined with

❖ **Removed text that was in line 279-285 in the Result section of original version**

~~DesNTD(AF-1)-ERα is almost equivalent to the native ERα isoform named ERα46, which was reported to be a weak transcriptional activator compared to ERα [21,22]. ERα46 is 4 amino acids longer than desNTD(AF-1)-ERα at its N-terminus. As an E2-inducible activator, ERα46 has been reported to be 20–100% as efficient as full-length ERα in assays using various cells and promoter reporter genes [21,22]. In the present study, we used ordinary firefly luciferase reporter plasmid pGL3, which contains an estrogen response element repeated three times (3×ERE).~~

❖ **The original text in line 611-616 in the Discussion section of original version**

Inquiry into the intrinsic functional role of ERα-NTD(AF-1)

As described above, desNTD(AF-1)-ERα is almost equivalent to the native ERα isoform ERα46 [21,22]. It should be noted, however, that ERα46 is a rather weak transcriptional activator; as an E2-inducible activator, it is 20–100% as efficient as full-length ERα. Recently, ERα46 was reported to be involved in the growth and metastasis of breast cancer cells [36]. Thus, what is its physiological role in breast cancer cells?

⬇

❖ **Newly combined and condensed text between the sentences in line 279-285 in the Results section and the sentences in line 611-616 in the Discussion section of revised version**

Inquiry into the intrinsic functional role of ERα-NTD(AF-1)

DesNTD(AF-1)-ERα is almost equivalent to the native ERα isoform named ERα46, which was reported to be a weak transcriptional activator compared to ERα [34,35]. ERα46 is 4 amino acids longer than desNTD(AF-1)-ERα at its N-terminus. As an E2-inducible activator, ERα46 has been reported to be 20–100% as efficient as full-length ERα in assays using various cells and promoter reporter genes. Recently, ERα46 was reported to be involved in the growth and metastasis of breast cancer cells [36]. Thus, what is its physiological role in breast cancer cells?

✜ As a result of this editing, the following changes have occurred.

(i) References No. 21 and 22, [21,22], were changed to No. 34 and 35, [34,35], respectively. With this, continuing references No. 23~35 were brought forward by two each, providing new numbers of 21~33.

(ii) The sentence in the old Result section, namely, "In the present study, we used ordinary firefly luciferase reporter plasmid pGL3, which contains an estrogen response element repeated three times (3×ERE)" was completely eliminated because of its insignificance or irrelevance for the discussion there.

(iii) The rest of sentences in the old Result section, namely, "DesNTD(AF-1)-ERα is almost equivalent to the native ERα isoform named ERα46, which was reported to be a weak transcriptional activator compared to ERα [21,22]. ERα46 is 4 amino acids longer than desNTD(AF-1)-ERα at its N-terminus. As an E2-inducible activator, ERα46 has been reported to be 20–100% as efficient as full-length ERα in assays using various cells and promoter reporter genes," was moved to and placed in the Discussion section to replace the sentences "As described above, desNTD(AF-1)-ERα is almost equivalent to the native ERα isoform ERα46 [34,35]. It should be noted, however, that ERα46 is a rather weak

transcriptional activator; as an E2-inducible activator, it is 20–100% as efficient as full-length ERα." The content is almost equivalent to each other, although the former is slightly minute.

As a whole, we believe that all these changes make the discussion on the ERα isoform ERα46 to be much clearer.

---

6. PLOS authors have the option to publish the peer review history of their article (what does this mean?). If published, this will include your full peer review and any attached files.

If you choose "no", your identity will remain anonymous but your review may still be made public.

Do you want your identity to be public for this peer review? For information about this choice, including consent withdrawal, please see our Privacy Policy.

Reviewer #1: No

Answer: We have no comment on this matter.
